# Oxygen-releasing biomaterials for chronic wounds breathing: From theoretical mechanism to application prospect

**DOI:** 10.1016/j.mtbio.2023.100687

**Published:** 2023-06-02

**Authors:** Yu He, Qiang Chang, Feng Lu

**Affiliations:** Department of Plastic and Reconstruction Surgery, Nanfang Hospital, Southern Medical University, 1838 North Guangzhou Avenue, Guangzhou, Guangdong Province, 510515, China

**Keywords:** Chronic wounds, Hypoxia, Oxygen, Biomaterials, Diabetic wounds

## Abstract

Chronic wounds have always been considered as “gordian knots” in medicine, in which hypoxia plays a key role in blocking healing. To address this challenge, although tissue reoxygenation therapy based on hyperbaric oxygen therapy (HBOT) has been performed clinically for several years, the bench to bedside still urges the evolution of oxygen-loading and -releasing strategies with explicit benefits and consistent outcome. The combination of various oxygen carriers with biomaterials has gained momentum as an emerging therapeutic strategy in this field, exhibiting considerable application potential. This review gives an overview of the essential relationship between hypoxia and delayed wound healing. Further, detailed characteristics, preparation methods and applications of various oxygen-releasing biomaterials (ORBMs) will be elaborated, including hemoglobin, perfluorocarbon, peroxide, and oxygen-generating microorganisms, those biomaterials are applied to load, release or generate a vast of oxygen to relieve the hypoxemia and bring the subsequent cascade effect. The pioneering papers regarding to the ORBMs practice are presented and trends toward hybrid and more precise manipulation are summarized.

## Graphical abstract

**Figure:**
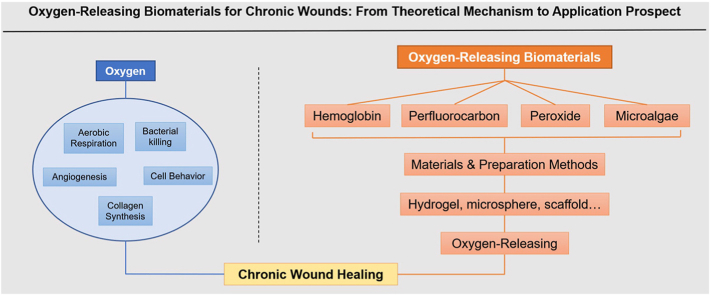

## 1 Introduction

In medicine, chronic wounds remain a persistent challenge, considering the devastating impact on the quality of individual life, the heavy economic burden to the medical system worldwide, and the growing number of patients annually [1,2]. In recent years, clinical and basic research has focused on the principles of delayed healing and efficient treatment of chronic wounds to address this challenge [3]. Notably, hypoxia has aroused marked interest as a pivotal factor in manipulating chronic wounds during primary healing. From the early stages of wound healing, the wound area receives insufficient blood perfusion owing to local microcirculation disturbances and suppressed angiogenesis. Meanwhile, the elevated metabolism in the wound area increases oxygen demand substantially, which further aggravates the hypoxia of tissue cells, and, in turn, hinders critical physiological processes such as bacterial killing, cell survival, and proliferation along with collagen deposition, collectively impeding wound healing outcomes [4].

Therefore, tissue reoxygenation therapy has opened an intuitive avenue for chronic wound closure. For instance, hyperbaric oxygen therapy (HBOT) and topical oxygen therapy (TOT) have afforded valuable adjuvant treatments in clinical practice for decades [[5], [6], [7]]. However, their widespread utilization has been hindered by high treatment costs, inconvenience, uncertain long-term efficacy, inconsistent clinical evaluations, and a series of complications [8,9]. Recently, integrated biomaterial platforms, such as hydrogel combined with various oxygen-releasing substances, have emerged, facilitated by the remarkable manipulability of hydrogel along with the loading capacity of functional molecules; these oxygen-releasing biomaterials (ORBMs) with specific functions have inspired a new strategy for the reoxygenation therapy in wound healing and skin regeneration [10].

## 2 Chronic wounds and oxygen

### 2.1 Why hypoxia?

Chronic wounds, primarily represented by diabetic wounds, pressure ulcers, and venous ulcers, are defined as wounds that fail to heal after more than 4 weeks of conventional wound-care treatment and exhibit no apparent healing tendency in the absence of external intervention [11]. In chronic wounds, hypoxia can be attributed to various pathological factors, including microcirculation disturbance, owing to local vascular injury or vascular sclerosis and persistent inflammatory response, bacterial infection, and fibrosis, which may also suppress new blood vessel formation [12]. In addition, the increased pH in the wound area also leads to the decreased oxygen-releasing performance of hemoglobin (Hb) [13]. Moreover, wound healing involves a series of physiological processes related to tissue repair and regeneration, which are highly active in oxygen metabolism. According to several studies, the wound area remained relatively hypoxic despite supplemental O_2_ treatment [14,15]. Under the contradictory dilemma of decreased oxygen supply and increased oxygen demand, the vicious “hypoxia cycle” cannot be broken spontaneously once formed, thereby restraining wound healing in the chronic inflammatory phase without progress. In normal tissues, oxygen only tends a distance of about 64 ​μm from the capillaries [16]. In skin wounds, the stratum corneum, exudate on wound surfaces, and eschar scars are more impermeable to oxygen [4]. In chronic wounds, oxygen partial pressure (pO_2_) ranges between 5 and 20 ​mmHg, compared that of 35–50 ​mmHg in normal skin tissue [4,17]. In addition, it should be noted that the pO_2_ was found to drop to 0–10 ​mmHg in the dermal wound center. These physiological and pathological mechanisms suggest that hypoxia governs the existence and severity of chronic wounds.

### 2.2 The importance of oxygen for wound healing

The importance of oxygen can be equated to the “source of life”. The survival, proliferation, and various critical physiological activities of human tissues and cells are facilitated by oxygen. Wound healing is no exception to either energy synthesis, cell function or angiogenesis.

#### 2.2.1 Coupled with aerobic respiration to generate ATP for energy

The most important role of oxygen is to participate in aerobic respiration, oxidizing nutrients to generate energy in the form of ATP for a series of physiological processes such as cell survival, proliferation, migration and differentiation, as well as transmembrane transport and signal transduction. Under insufficient oxygen conditions, tissue cells will switch to metabolic reprogramming, known as the “Warburg Effect”, inducing a change to the mode of anaerobic respiration, which results in inefficient nutrient utilization and metabolic acidosis owing to lactic acid accumulation [18,19]. Furthermore, in the case of chronic and severe hypoxia, cells will directly undergo autophagy, apoptosis, or necrosis [20,21].

#### 2.2.2 Enhancing neutrophil bactericidal function and antibiotic efficacy

Bacterial infection and colonization are frequently accompanied by chronic wounds, resulting in an extensive response and persistent inflammatory phase. Moreover, intractable bacterial biofilms may form and severely hinder wound healing [22,23]. Under sufficient pO_2_, neutrophils produce a considerable amount of reactive oxygen species (ROS) to induce the oxidative killing of bacteria through mitochondria and NADPH oxidase, a classic process called “respiratory burst” [24]. ROS are metabolic oxygen derivatives, mainly including superoxide anions (·O_2_^−^), hydroxyl radicals (·OH), hydrogen peroxide (H_2_O_2_), and singlet oxygen (^1^O_2_), with levels correlating directly with pO_2_. ROS exhibit potent oxidative capability and act as signaling molecules in various pathways, including chemotaxis and immune cells activations [25]. It has been reported that the bactericidal efficiency of neutrophils is proportional to pO_2_. For instance, half-maximal oxidant production of neutrophils occurred in the range of 45–80 ​mmHg pO_2_, reaching a maximal level when higher than 300 ​mmHg [26]; however, the bactericidal function of neutrophils was markedly impaired when pO_2_ was less than 40 ​mmHg [27]. In addition, several antibiotics such as azithromycin, use oxygen and its metabolic derivatives as reaction substrates, and hypoxia can directly attenuates the sterilization efficiency of these antibiotics. In general, adequate pO_2_ in the wound area is essential for clearance of bacterial infections.

#### 2.2.3 Promoting collagen cross-linking and deposition

As one of the most important components of the extracellular matrix (ECM), collagen, which is mainly synthesized and secreted by fibroblasts, is indispensable for wound healing. Exposure to chronic hypoxia was shown to negatively impact fibroblast activity and lead to a 3.1-fold decrease in the relative expression of transforming growth factor-beta1 (TGF-β1), which is responsible for the essential transcription of the procollagen gene [28].Furthermore, oxygen is vital for the entire collagen synthesis process, from the initial assembly to deposition as mature ECM. During collagen synthesis, oxygen-dependent enzymes, such as prolyl hydroxylase and lysyl hydroxylase, utilize oxygen as a basic substrate. Oxygen is also required for the cross-linking of procollagen into mature triple-helical collagen. Reportedly, the rate of collagen synthesis and working efficiency of corresponding enzymes are positively correlated with pO_2_. Furthermore, half-maximal collagen synthesis has been documented at a pO_2_ of 20–25 ​mmHg, with the maximum occurring at levels approaching 250 ​mmHg [14,[29], [30], [31]]. Accordingly, a sufficient level of pO_2_ can improve wound healing by promoting ECM formation.

#### 2.2.4 Promoting angiogenesis

It is well-known that a hypoxic microenvironment increases the expression of hypoxia-inducible factor (HIF-1), one of the target gene promoting the expression of vascular endothelial growth factor (VEGF), thereby facilitating angiogenesis [32,33]. However, although the process of angiogenesis can be temporarily stimulated by hypoxia, it cannot be maintained during chronic and persistent hypoxia. To date, the expression and effects of HIF-1 remain inconclusive in chronic wounds. In the conventional regulatory pathway of HIF-1, respective amino acid residues are oxidized by prolyl hydroxylase (PHL) under normoxic conditions, resulting in ubiquitination-mediated degradation. Whilst Sunkari and colleagues showed that HBOT could also stabilize and activate HIF-1 to increase expression levels of VEGF and stromal cell-derived factor 1(SDF-1), there promoting cellular proliferation [34]. Blocking HIF-1 expression can significantly inhibit wound healing when using HBOT to treat chronic diabetic wounds, indicating that HBOT and HIF-1 afforded a synergistic effect on wound healing. The authors speculated that HBOT activated the Hsp90 protein by increasing ROS generation, stabilizing HIF-1 and protecting it from degradation [35]. Another study has found that HBOT could upregulate the expression of VEGF while downregulate tumor necrosis factor (TNF)- α expression, thereby promoting angiogenesis, facilitating epithelialization and accelerating wound healing [36]. Furthermore, during the specific process of angiogenesis, oxygen can promote the growth of the vascular sprout matrix and the migration of vascular endothelial cells [37].

#### 2.2.5 Inducing cell migration and differentiation

As an important and sensitive factor of the cellular microenvironment, pO_2_ can regulate various cell behaviors along with wound healing acceleration through metabolic reprogramming and signaling pathways mediated by HIF-1 and ROS [38]. Hyperoxia can downregulate immune cell activity by reducing TNF-α expression, thereby alleviating the chronic inflammation response [36], and mild hyperbaric oxygen treatment in aged mice was shown to activate epidermal basal cell proliferation [39]. As described above, hyperoxia can positively impact the SDF-1-CXCR4 axis, which promoted the directed migration of stem cells and fibroblasts to the wound area, thereby accelerating wound healing [34,40]. In addition, it has been reported that under hyperoxia conditions, fibroblasts can be induced to differentiate into myofibroblasts, which can facilitate wound healing by generating tension and enhancing contraction [41]. Furthermore, oxygen can increase the expression of matrix metalloproteinases (MMPs), promoting the rearrangement and maturation of ECM [42].

**Fig. 1:**
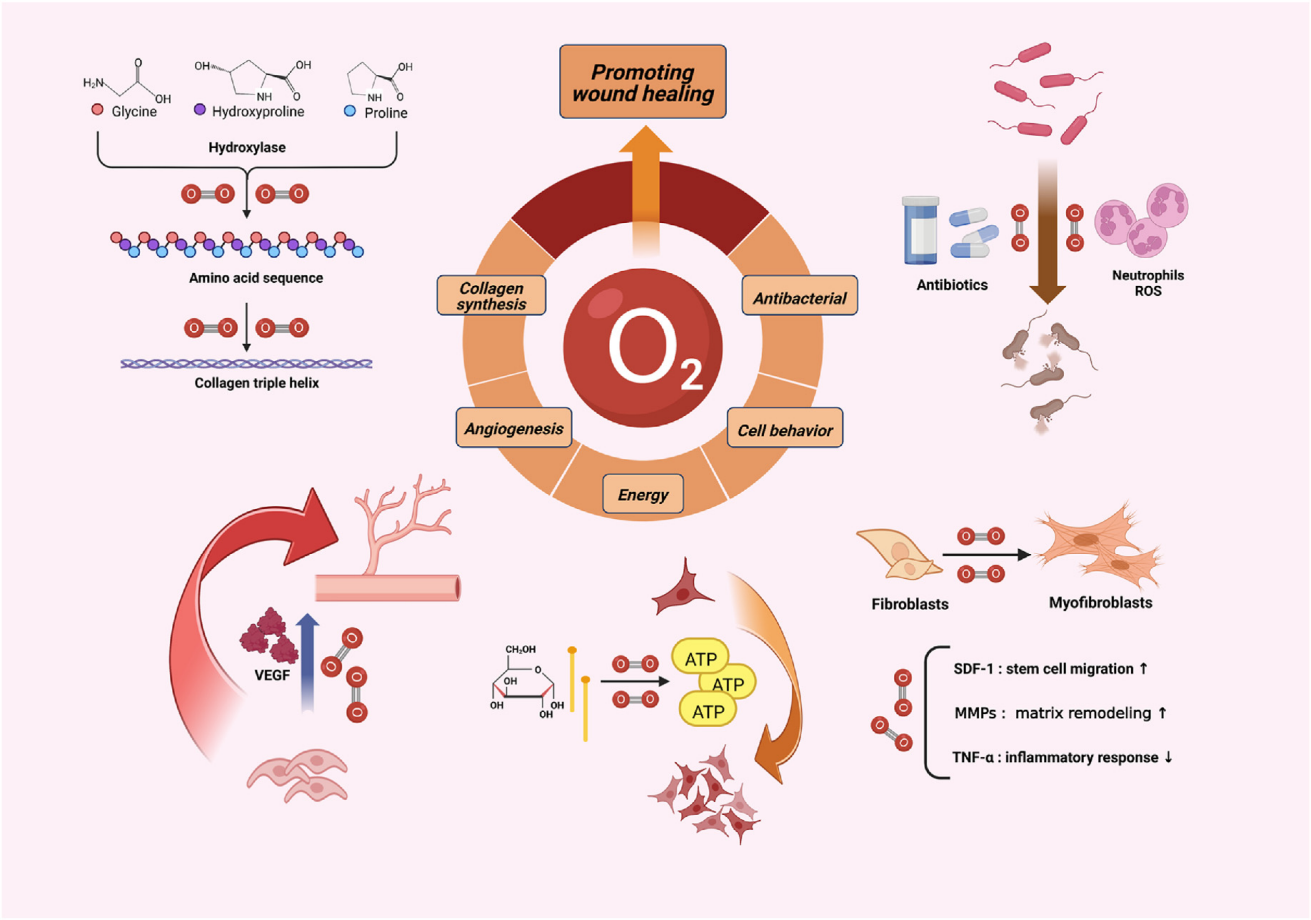
Schematic Overview of O_2_ Function in Wound Healing. Figure created with Biorender.com.

Based on the above findings, it can be suggested that oxygen can promote wound healing through diverse mechanisms (Fig. 1). PO_2_ is an important factor that impacts the microenvironment of tissue cells, although the optimal pO_2_ required for various physiological activities tends to differ [43]. In addition, it should be noted that, like the two sides of a coin, the roles of hypoxia and hyperoxia during wound healing should be analyzed dialectically rather than based on dogmatic definitions [44]. Table 1 summarizes several relevant and critical pO_2_ values related to wound healing.

**Table 1:** Critical pO_2_ Values for Wound Healing.

Micro environment or physiological process	Oxygen partial pressure (pO_2_/mmHg)	Ref.
Atmosphere	159 (20.4%, 291.4 ​mg/L)	–
Artery	95–100	–
Normal skin tissue	35–50	[4,17]
Chronic wounds	5–20	[4,17]
Dermal wounds center	0–10	[4,17]
Neutrophil bactericidal function	45-80 half-maximum; 300 maximum	[26,27]
Collagen synthesis	20-25 half-maximum; 250 maximum	[[29], [30], [31]]

### 2.3 Current strategies for reoxygenation therapy

#### 2.3.1 HBOT

Reoxygenation therapy is the restoration of normal oxygenation in hypoxic tissues using corresponding methods. HBOT is a therapeutic strategy that has been approved for decades and is routinely used in clinical practice. During HBOT, pure oxygen is inhaled in a specific hyperbaric chamber to increase blood pO_2_ and improve tissue oxygenation [45]. Since the American Diabetes Association endorsed HBOT for treating recalcitrant diabetic foot ulcers in 1999, it has been widely used and explored for treating chronic wounds [6,46]. According to several clinical and experimental studies, HBOT can promote the healing of chronic wounds to a certain extent by various mechanisms, such as enhancing the local pO_2_, reducing the inflammatory response, promoting the secretion of VEGF to enhance angiogenesis, and mobilizing bone marrow mesenchymal stem cells to migrate to the wound area [[47], [48], [49], [50]]. However, it is worth noting that tissue reoxygenation via HBOT depends on the functioning vascular system. Although the pO_2_ of blood increases, it can only reach wound sites through blood transport, while many chronic wounds suffer the vascular insufficiency. Some retrospective analyses have shown that HBOT was only effective in the short term for improving wound healing, whereas long-term efficacy remains unclear and inconsistent in clinical evaluation [6]. In addition, HBOT may be associated with barotrauma, oxygen toxicity, poor cost-effectiveness, and general patient compliance. Nevertheless, HBOT is still advisable for the clinical treatment of chronic wounds, and provides some clinical evidence and research ideas for reoxygenation therapy.

However, it is worth mentioning that HBOT is virtually an adjuvant therapy in the whole systematic treatment of wounds which is called as TIME principle: tissue debridement, infection control, moisture balance, and edges of the wound. Therefore, in addition to HBOT, it is necessary to use ideal dressings whose characteristics should include: (1) absorb excessive exudates; (2) control the moisture in the wound bed; (3) possess good mechanical stability; (4) have great gases transmission; (5) protect from microorganism colonization and infections; (6) be non-toxic, biocompatible, and biodegradable [51].

#### 2.3.2 TOT

TOT is a further improvement of reoxygenation therapy that involves supplementing oxygen to hypoxic sites, specifically with a particular oxygen delivery system. Currently, there are three main TOT devices clinically used for treating chronic wounds: (1) those providing continuous delivery of oxygen (Natrox ®); (2) those providing constant low-pressure in a contained chamber (OxyCare ®); (3) those that are cyclically pressurized and humidified in a contained chamber (TWO_2_ ®) [7]. Although these devices vary considerably from mechanism to operation method, they can effectively promote diabetic wound healing to some extent [52,53]. Interestingly, a previous report found that direct jetting of gaseous oxygen to infected wound sites at a specific frequency could promote healing [54]. The processes occurring in TOT are similar to those in the whole-body treatment, causing the intensification of oxygen diffusion and increased oxygen partial pressure in the tissues due to hydration. TOT is much cheaper, easier to use, available to patients, and based on the same physical phenomenon [55,56]. In addition, compared with HBOT, TOT could reduce side effects and the economic burden on patients to a certain extent, gaining better systemic benefits under local treatment.

#### 2.3.3 ORBMs

Notably, the aforementioned reoxygenation therapies only provide simplex oxygen and are mostly employed as complementary treatments. As a novel concept, ORBMs involve the introduction of specific materials to finely regulate the oxygen release kinetics in terms of time and rate, facilitating the exploration of the deep regulatory mechanism of reoxygenation therapy. As a notable advantage, ORBMs allow the loading of bioactive ingredients to accelerate wound healing synergistically. For example, various growth factors and stem cells can be co-packaged with ORBMs to construct a multifunctional oxygen-releasing platform. Thus, the superior benefit of reoxygenation therapy can be exerted by several positive factors, functioning in a synergistic paradigm to promote wound healing. In addition, most ORBMs are composed of cost-effective materials that allow convenient utilization, further broadening their clinical application potential in chronic wounds healing. Various ORBMs are detailed in the following sections.

## 3 Oxygen-releasing biomaterials

ORBMs can be categorized into two subtypes according to oxygen-releasing substances, ⅰ: oxygen-carrying substance that can preload and release oxygen molecules based on intrinsic characteristics, mainly including Hb and perfluorocarbons (PFCs); ⅱ: oxygen-generating substances that can generate oxygen through chemical reactions, mainly includes peroxides and photosynthetic microalgae. Gelation, emulsion evaporation, three-dimensional (3D) printing, and electrospinning have been used to combine these oxygen-releasing substances with various biomaterials to afford specific functional materials such as oxygen-releasing hydrogels and microspheres.

3.1

**Fig. 2:**
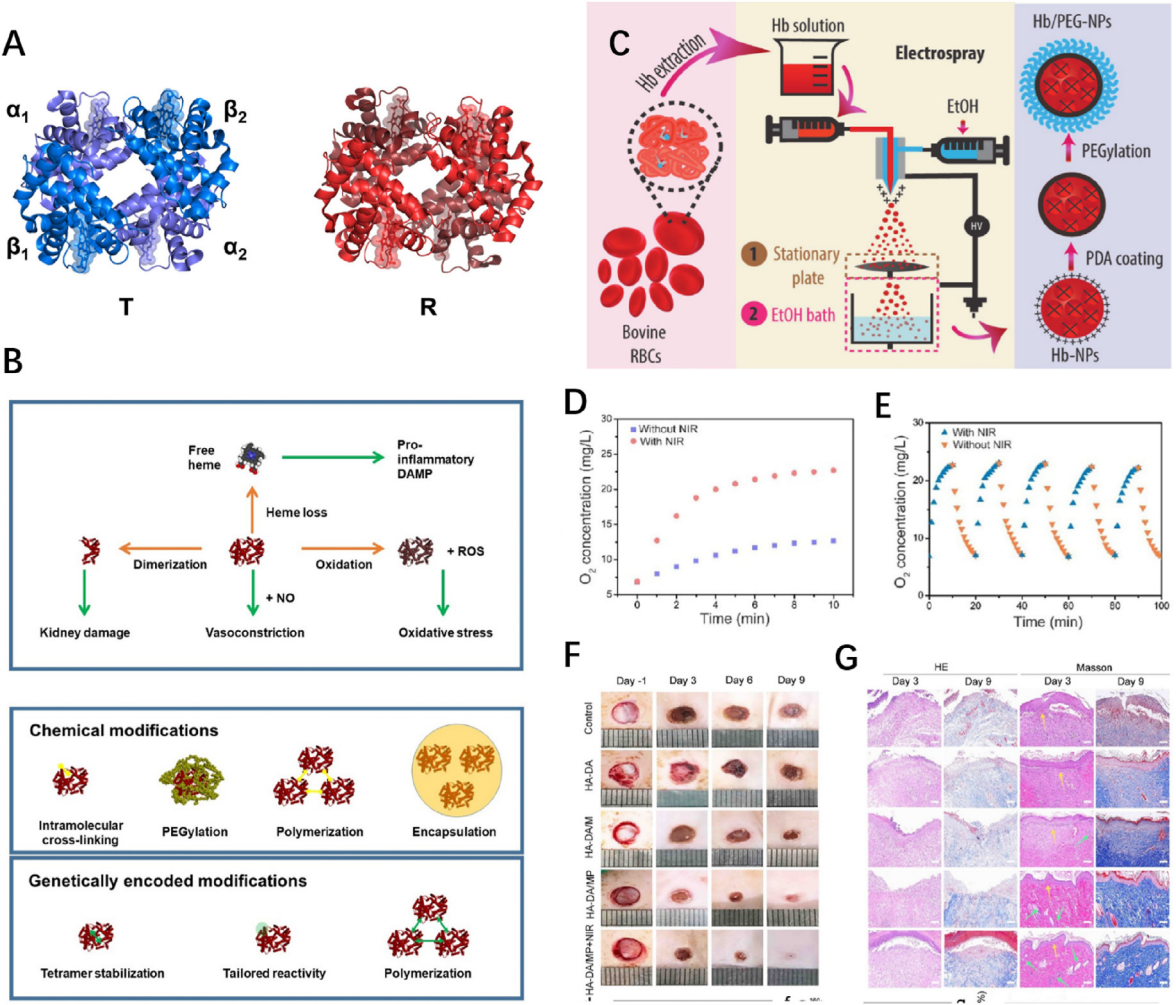
**Hb ORBMs. (A)** Overall quaternary structure of Hb: deoxygenated (T state) Hb (blue) and oxygenated (R state) Hb (magenta). **(B)** Potential toxic effects caused by free Hb and strategies for the development of HBOCs. **(C)** Illustration of the assembly process. **(D、E)** oxygen release by photothermal conversion and controllable oxygen delivery evaluation. **(F、G)** Images of wounds with different treatments on days-1, 3, 6, and 9. Hb, hemoglobin; ORBMs, Oxygen-releasing biomaterials; HBOCs, Hb-based oxygen carriers; PDA, polydopamine. Reproduced with permission.^[^ [58]^],^ [72,73] Copyright 2022, Elsevier. Copyright 2020, Wiley. Copyright 2022, American Chemical Society. (For interpretation of the references to color in this figure legend, the reader is referred to the Web version of this article.)

Hb is a macromolecular compound with four subunits (2α+2β), each possessing a globin polypeptide chain and a heme prosthetic group containing ferrous ions (Fe^2+^) that coordinate with oxygen molecules, endowing Hb the ability to carry oxygen (Fig. 2A) [57]. Given its attractive functions, a myriad of researchers has explored Hb as a potential oxygen carrier since the last century [58]. Although rare relative products have been clinically recognized and applied consistently to date, Hb has been deemed a potential oxygen carrier in clinics and either in clinic and laboratory settings, given merits of natural human origin and the inherent oxygen affinity.

Hb contains four different ligand binding sites: heme, exposed cysteine residues, DPG binding sites and Bohr residues [59]. Changes in any binding site can induce corresponding structural alterations in Hb, affecting the balance between the “T” (tense) and “R” (relaxed) states, subsequently resulting in altered oxygen affinity, called “allosteric effects” [57]. Under normal physiological conditions in the human body, the allosteric effect has a Hill coefficient approximating 3. The binding of O_2_ to the heme group increases the oxygen affinity of Hb. Meanwhile, endogenous ligands such as 2,3-diphosphoglyceride (2,3-BGP), proton (H^+^), carbon dioxide (CO_2_), and chloride reduce the oxygen affinity of Hb through the Bohr effect, thereby leading to oxygen release. In addition, exogenous drugs (e.g. bezafibrate) and environmental factors (e.g. elevated temperature) can also reduce the oxygen affinity of Hb [59]. Nevertheless, the precise mechanisms underlying this phenomenon remain unknown.

Moreover, Hb acts as a multifunctional molecule by regulating pH and maintaining the redox state. In addition, the prosthetic heme group functions as a nitric oxide (NO) synthase, monooxygenase and peroxidase, participating in related biochemical reactions [60]. What's more, following proteolytic cleavage, Hb breaks down to produce several biologically active compounds, including hemocidins, which exhibit antibacterial properties by participating in innate immune response and wound healing [61].

However, after exiting erythrocytes, free Hb molecules break down to dimers from tetramers, with high oxygen affinity yet no allosteric effect, which leads to the de-functionalization of oxygen-carrying. In addition, free Hb (with Fe^2+^) molecules are transformed into inactive methemoglobin (MetHb; with Fe^3+^), and the further self-oxidation of MetHb generates free radicals and causes subsequent oxidative damage, including excessive NO clearance, which triggers pathological vasoconstriction [62]. Furthermore, small Hb molecules of Hb may induce renal damage through blood circulation via pathological deposition in the kidneys [63].

Therefore, it is necessary to encapsulate and protect free Hb molecules during Hb-based oxygen carrier (HBOC) development. Several strategies have been implemented such as selecting different types of Hb or modifications, intermolecular cross-linking, and synthesis of protective encapsulation systems (Fig. 2B). For example, fetal Hb (HbF), which is more stable and less prone to oxidation, exhibits a higher oxygen affinity and superior oxygen-carrier capability than normal Hb [64]. Ozcelik et al. used Hb from a marine worm (named M101, developed by biotech company Hemarina) as an oxygen carrier with hyaluronic acid as a polymer matrix to synthesize a corresponding oxygen-releasing hydrogel [65]. Up to 156 oxygen molecules could be loaded by each M101 molecule, which exhibited an oxygen-carrying capacity nearly 40 times that of human Hb (4 of human Hb). The oxygen concentration steadily increased to 1 ​mg/L within 1 ​h of placing the hydrogel in the initial hypoxic medium. In addition, the p50 (pO_2_ at 50% saturation of Hb) and the allosteric effect of M101 at 37 ​°C were similar to those of human Hb [66]. Furthermore, the authors verified its anti-inflammatory effect and biocompatibility, indicating the substantial potential of M101 for human applications. Paciello et al. conjugated free amino and carboxyl groups in Hb to the surface of gelatin microspheres using conjugation agents N,N′-disuccinimidyl carbonate (DSC) or 1-ethyl-3-(3-dimethylaminopropyl)carbodiimide (EDC) to improve its stability [67]. Notably, the oxygen uptake/release performance of the microsphere varied with different conjugation strategies showed no significant difference. The microspheres could uptake O_2_ in the solution under normoxic conditions and release it under hypoxic conditions, with the stabilized O_2_ concentration within 24 ​h. Additional cyclic O2 uptake/release in the microcirculation test also demonstrated excellent performance for organ perfusion and tissue culture. Wang et al. prepared dextran-bovine Hb by covalently conjugating periodate-oxidized dextran with bovine Hb based on the dialdehyde method to protect the thiol part of Cys-93 (β), resulting in improved antioxidant capacity and prolonged the lifespan of Hb [68]. Moreover, the p50 of dextran-bovine Hb reached 9.86 ​mmHg when compared with that of bovine Hb under the same conditions (26.19 ​mmHg), which implied markedly improved O_2_ affinity and indicated its application potential as HBOC. Moreover, the conjugation process would inevitably impact on the physicochemical properties of Hb; hence, the fundamental oxygen-carrying activity of the heme group should be listed preferentially.

Inspired by the adhesion and antioxidant properties induced by the abundant catechol groups of polydopamine (PDA), Wang et al. prepared Hb-PDA nanoparticles (NPs) to stabilize Hb and maintain its physiological function. The excellent antioxidant properties and free radical scavenging ability of PDA could protect the encapsulated Hb by reducing the generation of non-functional MetHb to prolong the lifespan of Hb [69]. Furthermore, the function of NPs could be readily tuned by adjusting the pH and the dopamine/Hb mass during the polymerization process [70]. Centis et al. found that encapsulating Hb with polyethylene glycol (PEG) liposomes could double the O_2_ release time and equilibrium concentration [71]. Cell growth in fibrin hydrogels was promoted after 24 ​h under higher O_2_ conditions provided by PEGylated liposomes. Notably, particles < 100 ​nm can pass through capillaries, whereas particles >1 ​μm can be easily recognized and then phagocytosed by macrophages, both of which result in rapid clearance. Therefore, as a blood substitute in vivo or as an ORBM for wound healing, the size of NPs should be considered. Liu et al. formulated Hb/PDA NPs with tunable particle size by electrostatic spraying and further functionalized the PDA shell with PEG (Fig. 2C), resulting in size of approximately 400 ​nm [72]. Meanwhile, as one of the gold-standard strategies to circumvent the mononuclear phagocytosis system, surface PEGylation could further improve the biocompatibility of NPs with the immune system and prolong their functional lifetime.

The efficacy of HBOC in treating of chronic wounds has also gained popularity from in both academic and clinical. Li et al. designed a multifunctional oxygen-releasing platform based on a hyaluronic acid hydrogel containing photothermal MXene nanosheets and Hb [73]. The platform was rooted in the horseradish peroxidase activity of Hb, cross-linked with H_2_O_2_/HbO_2_ as an initiator system and supplemented with PDA as an antioxidant. MXene could convert energy from the irradiation of near-infrared (NIR) light into heat, thereby elevating the temperature of bulk hydrogel, which reduced oxygen affinity and triggered oxygen release; the local oxygen concentration increased from 6.87 to 23 ​mg/mL within 10 ​min following NIR exposure. Interestingly, the hydrogel could capture oxygen when the temperature decreased (Fig. 2D、E). In addition, the oxygen release property of the hydrogel remained unchanged even when the NIR on/off cycle was continuously performed five times over 100 ​min, further confirming the controllable and repeatable oxygen release capacity of the hydrogel. On applying the hydrogel to wounds of diabetic mice, the wound closure rate reached 98.8% on day 9th, when compared with that of the control group (90%) lacking NIR irradiation and the blank control group (60%) (Fig. 2F、G). Based on the observed findings, oxygen-releasing function of the hydrogel, along with other active components, significantly enhanced the healing of diabetic wounds by promoting the formation of the epidermis and new blood vessels, increasing collagen deposition, and exerting anti-oxidative and antibacterial effects.

Hunt et al. conducted a trial using a Hb spray (Granulox, Infirst Healthcare Ltd, London, UK) to treat patients with chronic wounds [74]. The Hb spray contained purified Hb, which served as the primary therapeutic agent, binding atmospheric oxygen and transporting it to the wound bed to aid diffusion, subsequently improving oxygen availability in the wound area. The patients were divided into two groups that typically underwent two dressing changes per week. In the Hb spray group, the patients used the spray at each dressing change until wound closure. At the end of the 26-week evaluation period, 45 of 50 (90%) patients in the treatment group showed complete wound healing, with a mean wound healing time of only 6.6 weeks, compared with 19 of 50 (38%) patients in the control group, exhibiting an average healing time of 11.4 weeks. In addition to the significantly accelerated wound healing, the Hb spray afforded substantial benefits in terms of reducing pain and exudate levels, as well as decreasing psychological anxiety and financial burden. Based on the experimental findings, the best results could be achieved by applying the Hb spray throughout the healing of chronic wounds, which might suggest that oxygen supplementation was required continuously during the entire healing phase. Moreover, premature discontinuation might cause the wound to revert to a hypoxic state, halting or worsening wound healing. Collectively, this clinical evaluation established positive conclusions and supported the inclusion of the Hb spray in standard wound-care regimens.

### 3.2 PFCs

**Fig. 3:**
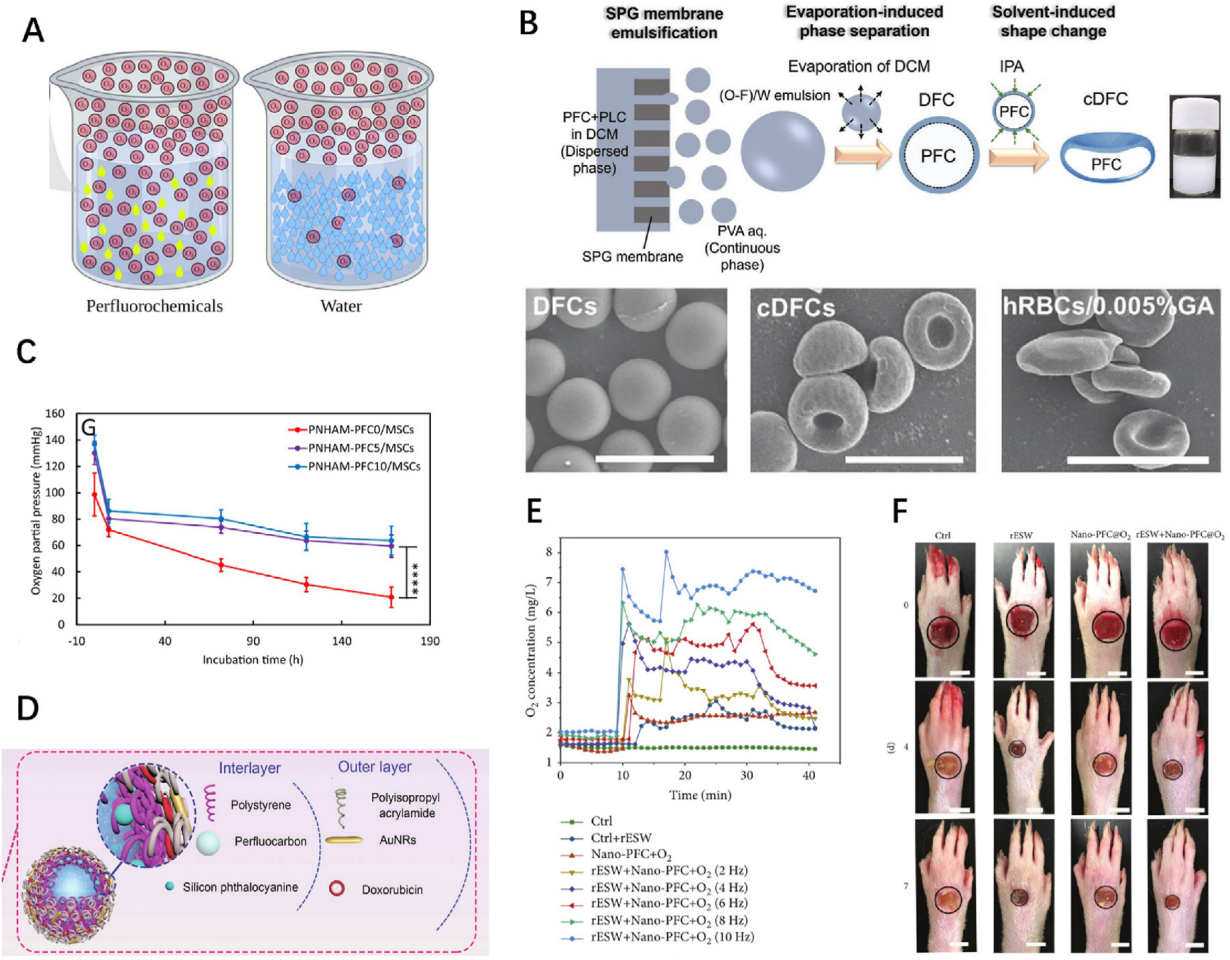
**PFC ORBMs. (A)** Illustration of the O_2_ solubility of PFC and water. **(B)** Upper: Schematic representation of the preparation process of concave-shaped microspheres. Lower: SEM images of the normal and concave-shaped microspheres. **(C)** PO_2_ of the hydrogels with different PFC content when incubated with cells. **(D)** Schematic illustration of the microsphere constituents. **(E)** Time-/dose-dependent changes of dissolved oxygen concentrations in deoxygenated pure water when treated with rESW for 25min. **(F)** Healing efficacy assessment of Nano-PFC for diabetic foot ulcers. ORBMs, Oxygen-releasing biomaterials; PFC, perfluorocarbon; rESW, radial extracorporeal shock wave; SEM, scanning electron microscopy; TEM, transmission electron microscopy. Reproduced with permission.^[^ [75]^],^ [80,81,84,85] Copyright 2021, Springer. Copyright 2022, Wiley. Copyright 2020, Elsevier. Copyright 2019, Hindawi.

PFCs are a class of compounds formed by replacing all hydrogen atoms with fluorine in hydrocarbon compounds. The abundant fluorine atoms endow PFCs molecules with increased polarity and further facilitate their hydrophobicity and lipophilicity [75]. In addition, the strong electronic attraction of fluorine atoms, accompanied by a relatively large intermolecular distance owing to the extremely weak interaction force between molecules, can afford PFCs the capacity to attract and accommodate numerous gas molecules through van der Waals forces. Therefore, PFCs can well-dissolve a wide spectrum of gases and are often used to store oxygen (Fig. 3A) [76]. As early as the 1980s, PFCs were examined as potential substitutes for human blood, and several experimental and clinical studies have been undertaken to date [77]. As a typical representation of PFCs, perfluorooctyl bromide (PFOB) is one of the most commonly used oxygen carriers in the biomedical field. PFOB is liquid at room temperature and can dissolve up to 50% of its volume, which is 125-fold that of water and 2.5-fold that of blood [77]. Moreover, PFOB exhibits excellent biocompatibility and chemical inertness. Owing to these characteristics, PFCs have also been examined as contrast agents for magnetic resonance imaging and as adjunctive therapeutic agents for photodynamic therapy [78,79].

Hydrophobicity and lipophilicity are primary considerations in preparing PFC biomaterials. Stable PFC emulsions can be prepared by encapsulating them in microparticles under amphiphilic emulsifiers. Fu et al. synthesized core-shell microspheres with oxygen-releasing properties using the Shirasu porous glass membrane emulsification technology after mixing a PFOB solution with a poly (lactide-*co*-caprolactone) (PLC) solution [80]. The particle shell was highly variable, forming a double concave disk with a similar structure and comparable size to red blood cells when immersed in isopropanol (Fig. 3B). The dissolved oxygen experiment further confirmed that prepared microparticles exhibited had similar physiological functions to red blood cells, considering oxygen release, which undoubtedly benefited from the PFC emulsions. Conversely, PFCs can be grafted into macromolecular compounds and further prepared into microspheres or hydrogels. Niu et al. grafted perfluorooctanoyl chloride (PFC) to poly (ethylene glycol) methacrylate (PEGMA), copolymerizing it with the hydrogel matrix *N*-isopropylacrylamide (NIPAAm) based on reversible addition-fragmentation chain transfer polymerization, thereby to preparing a hydrogel with a high oxygen storage capacity (Fig. 3C) [81]. The prepared hydrogel could maintain a the pO_2_ exceeding 60 ​mmHg even after 168 ​h of co-incubation with cells under 1% O_2_. Pritam et al. constructed a hydrogel dressing by grafting PFC onto methacrylamide chitosan [82]. After loading with sufficient oxygen, the hydrogel dressing could continuously release oxygen for up to 48 ​h, with a functional lifespan similar to that of a clinical dressing change. Furthermore, metabolomic analysis experiments demonstrated that the hydrogel prepared could accelerate wound healing by promoting epithelialization and collagen synthesis. It is worth mentioning that the hydrophobicity of PFC can also be exploited. Inspired by the low adhesion of the lotus leaf, Li et al. applied a PDA coating on medical gauze, followed by surface modification using PFC to obtain low adhesion via hydrophobic interactions [83]. Based on the experimental findings, the gauze showed limited adherence to the newly formed wound tissues, significantly weakening the tearing and destruction effect during the dressing change, and possibly accelerating wound healing to a certain extent.

Under normal circumstances, excessively dissolved oxygen in PFC gradually escapes through gradient diffusion. Given that PFCs mainly enhance the binding of gas molecules through physical force, the application of external thermodynamic disturbance factors, such as elevated temperature and cavitation effects of ultrasonic energy, can promote the dissolution of gas molecules. These characteristics can provide novel strategies for designing oxygen-releasing functions of materials. For example, Zhang et al. encapsulated PFC in functionalized bilayer polymers with gold nanorods (a photothermal conversion material) deposited on its outer shell (Fig. 3D) [84]. Irradiating with a 980 ​nm laser could increase the particle temperature, causing the escape of oxygen dissolved in PFC, which increased the oxygen content in specific positions and exerted a corresponding therapeutic effect. The authors referred to the particle as an “Oxygen Bomb” metaphorically, based on its functional properties. Using human serum albumin as a biomacromolecular emulsifier, Wang et al. prepared a nano-scale PFC emulsion and verified its responsive oxygen-releasing property under the action of radial extracorporeal shock wave (rESW) [85]. Subsequent *in vitro* and in vivo experiments revealed that rESW could trigger oxygen-saturated PFCs to release oxygen in the wound area and significantly accelerate the healing of diabetic foot ulcers (Fig. 3E、F). Another method to control the oxygen release kinetics involves the strategic application of emulsifiers. The type, thickness, and porosity of the emulsifier shell can distinctly impact the oxygen permeability, which may provide appropriate designs for yielding PFC emulsions with sustained-release properties. Jalani et al. added graphene oxide (GO) as a surfactant to PFC emulsions, endowing stability and barrier properties to prepared droplets [86]. The layered superposition property of GO hindered the rapid escape of encapsulated oxygen molecules, resulting in a mild sustained-release within at least 60 ​min, with superior performance observed on increasing the GO content. These findings provide an additional feasible solution to overcome the rapid release and poor stability of the PFC oxygen delivery system.

To treat chronic wounds, Lee et al. developed a chitosan-based heterogeneous composite hydrogel, encapsulating PFC emulsions and NPs loaded with epidermal growth factor (EGF) [87]. Considering the prepared hydrogel, the PFC-dissolved oxygen could enhance the effect of EGF in promoting cell proliferation. Based on the observed experimental findings, the wound healing efficiency was reduced, regardless of the absence of PFC emulsions or EGF. Under the synergistic effect of PFC and EGF, the wound healing in diabetic rats was significantly accelerated. The inflammatory reaction in the wound area was reduced, accompanied by elevated collagen content and maturity and complete re-epithelialization, the wound closure rate reached 95% on day15, which was more than 10% higher than that of both gauze- and HeraDerm (commercial dressing) -dressed wounds.

### 3.3 Peroxide

Peroxides are compounds with unstable peroxy bonds that can be broken under certain conditions, causing the original compound to decompose and generate oxygen. Based on their different physical properties, peroxides are divided into liquid peroxides, mainly hydrogen peroxide (H_2_O_2_), and solid peroxides, including calcium peroxide (CPO; CaO_2_), magnesium peroxide (MPO; MgO_2_), and sodium percarbonate (SPO; 2Na_2_CO_3_·3H_2_O_2_).

#### 3.3.1 H_2_O_2_

**Fig. 4:**
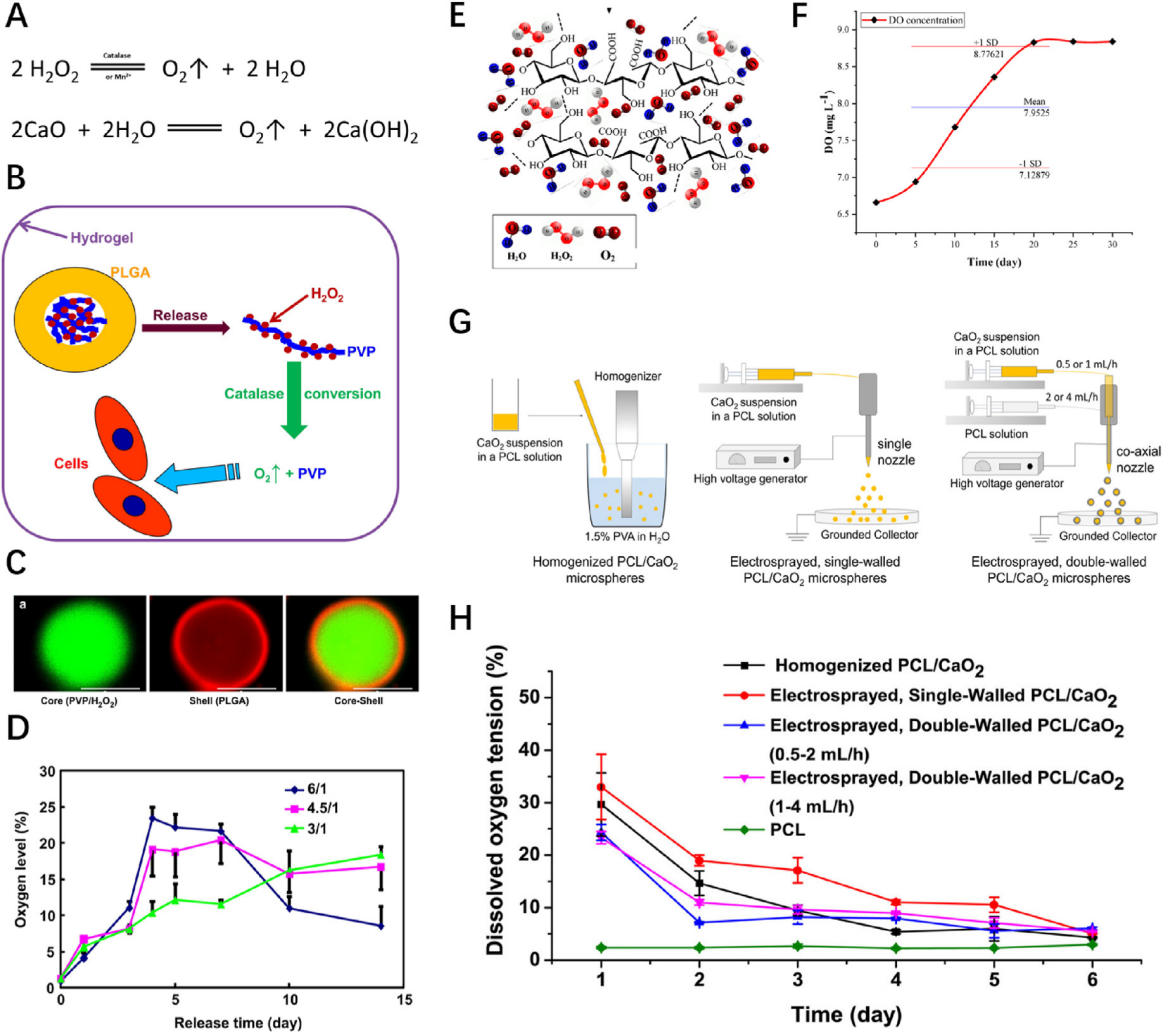
**Peroxide ORBMs. (A)** Oxygen-generating mechanisms of peroxides. **(B)** Design of oxygen-releasing microspheres to augment cell survival and differentiation. **(C)** Confocal images of core-shell H_2_O_2_-releasing microspheres. **(D)** Oxygen release kinetics of the H_2_O_2_-releasing microspheres with different H_2_O_2_/VP ratios. **(E)** Trapping/gradual releasing of oxygen from BC. **(F)** Oxygen release over time of BC/H_2_O_2_. **(G)** Illustration of the three methods used to fabricate PCL/CaO_2_ composite microspheres. **(H)** Time profiles of oxygen tension in PBS incubated with microspheres fabricated through different methods over 6 days. BC, bacterial cellulose; GelMA, gelatin methacrylate; ORBMs, oxygen-releasing biomaterials; PBS, phosphate-buffered saline; PCL, polycaprolactone; PLGA, poly (lactic-*co*-glycolic acid); SEM, scanning electron microscopy. Reproduced with permission.^[^ [92]^],^ [93,101] Copyright 2012, Elsevier. Copyright 2020, 2021, Wiley.

H_2_O_2_ is liquid at room temperature, with a boiling point of 150.2 ​°C, and miscible in water in any proportion. Owing to the instability of its peroxy bonds, H_2_O_2_ can decompose spontaneously to generate water and oxygen through a disproportionation reaction, which can be accelerated by elevated temperatures, pH, and catalysts such as metal ions or catalase (Fig. 4A). A 3% H_2_O_2_ solution is commonly used in clinical practice for wound disinfection. In addition, H_2_O_2_ is a ROS metabolized by normal cells (O_2_—NADPH--^.^O_2_^−^--SOD--H_2_O_2_), subsequently participating in various physiological activities as signal transduction molecules and metabolic regulators [88]. Moreover, the effect of H_2_O_2_ on cells is dose-dependent [89]. At low concentrations, H_2_O_2_ can promote cell proliferation and movement and regulate cell cycle through the Cdk–Prx–APC/C axis; at concentrations exceeding 0.4 ​mM, it acts as a broad-spectrum apoptosis inducer [90]. Therefore, direct contact may lead to undesirable consequences, and encapsulating H_2_O_2_ within a specific tissue cell-isolated space may be a suitable strategy when used as an oxygen source.

One common strategy encapsulation strategy involves the use of a macromolecular polymer matrix with good biocompatibility as the casing layer for the H_2_O_2_ solution. The thickness, porosity, oxygen permeability, and other properties of the encapsulation layer significantly impact oxygen-release release kinetics. Syed et al. fabricated an oxygen-releasing micro-system by dispersing poly (lactic-*co*-glycolic acid) (PLGA) copolymer microspheres encapsulating H_2_O_2_ in sodium alginate hydrogels immobilized with catalase [91]. The microsphere-released H_2_O_2_ underwent catalase-mediated decomposition in the matrix to generate oxygen. Notably, the oxygen release rate was significantly decreased with increasing sodium alginate hydrogel content, which was attributed to enhanced cross-linking degree and reduced porosity, restricting the diffusion of oxygen molecules. Li et al. prepared core-shell oxygen-generating microspheres by encapsulating a compound formed by reacting H_2_O_2_ with polyvinylpyrrolidone (PVP) in PLGA (Fig. 4B–D) [92]. The complexation of the macromolecular compound PVP could markedly limit H_2_O_2_ release, thereby endowing the microspheres with excellent H_2_O_2_ sustained-release performance in direct proportion to the PVP content. In Dulbecco's phosphate-buffered saline solution supplemented with catalase, the microspheres continued to release oxygen steadily for at least two weeks, maintaining an oxygen concentration of >15% by day 14. Sarkandi et al. soaked dried bacterial cellulose (BC) membranes in a 3% H_2_O_2_ solution for 5 ​h to induce the formation of BC/H_2_O_2_ complexes [93]. The dense nanostructure formed by BC cross-linking restricted the oxygen molecules generated by H_2_O_2_ decomposition. BC/H_2_O_2_ complexes dissolved in distilled water steadily released oxygen for at least 20 days, uniformly increasing the dissolved oxygen content to 8.8 ​mg/L (Fig. 4E、F).

Interestingly, direct encapsulation of H_2_O_2_ is not the only viable preparatory method. H_2_O_2_ can also be generated *in situ* using relevant reaction systems and enzymatically decomposed to release oxygen. Huber et al. prepared a succinyl chitosan (SC)-carboxymethyl cellulose (CMC) hydrogel system, in which CMC was degraded by cellulase into cellobiose to generate H_2_O_2_ *in situ* under the action of cellobiose dehydrogenase immobilized on chitosan [94]. Although the study mainly investigated the role of H_2_O_2_ produced by the dual-enzyme system in promoting the healing of infected wounds, the findings can afford inspiration for future investigations: if catalase is added to convert H_2_O_2_ into oxygen, the results might be even more positive.

For chronic wound therapy, Wang et al. incorporated glucose oxidase (GOx) and catalase into polydopamine/acrylamide (PDA/AM) hydrogels to promote the healing of diabetic wounds [95]. Considering the prepared hydrogel, GOx could oxidize the excessive glucose in the wound area to gluconic acid and H_2_O_2_ with the assistance of atmospheric oxygen molecules, followed by the breakdown of H_2_O_2_ into oxygen using catalase. Although there was no net oxygen generation in the whole reaction system, the dissolved oxygen experiments showed that the hydrogel increased the oxygen content of the solution by 10 times, up to 18.8 ​mg/L, compared with that in the control group. As a possible explanation, the double-enzyme system could be equivalent to transporting atmospheric oxygen to the wound area through cascade reaction, thereby increasing the local oxygen content. In further animal experimentation, the oxygen-generating function and other hydrogel-mediated effects were shown to increase collagen deposition and the formation of new blood vessels, significantly promoting diabetic wound healing.

#### 3.3.2 Solid peroxides

Following contact with water, solid peroxides are decomposed to generate H_2_O_2_, O_2_ and their corresponding hydroxides (Fig. 4A). Therefore, it is particularly important to comprehensively clarify the water-splitting behavior of solid peroxides if selected as a chemical oxygen source. Studies have found that the solubility of solid peroxides and the solution pH are the main factors that impact the generation of H_2_O_2_ and O_2_ [96]. Among all available solid peroxides, CPO is the most commonly used owing to its availability, while MPO has the longest oxygen release time and the lowest cytotoxicity at the same dose. Wang et al. conducted a kinetic study to assess the water-splitting behavior of CPO [97]. Interestingly, the results revealed that the CPO-induced production of H_2_O_2_ and O_2_ involved two independent reactions, rather than the traditional concept of producing H_2_O_2_, followed by the subsequent production of O_2_ via its decomposition. Moreover, increasing the pH and reaction temperature could incline the two mutually antagonistic sub-reactions in the direction of oxygen generation, thereby increasing oxygen production.

The predominant challenge experienced with the use of solid peroxides as chemical oxygen sources is the toxic side effects caused by by-products such as metal ions, increased pH, H_2_O_2_ and other ROS. A common strategy is the addition of antioxidants such as catalase and buffer solutions. For example, Wang et al. introduced the antioxidant ascorbic acid (vitamin C) into antimicrobial nanofibers of polycaprolactone (PCL) incorporating CPO, which alleviated the robust oxidative stress to a certain extent, reducing toxicity to cultured cells [98].

Accordingly, direct contact between solid peroxides and the wound area should be avoided. For application as ORBMs, the issues concerning encapsulation and isolation need to be resolved. Jeon et al. directly mixed alginate and CPO solutions, and the dissociated divalent calcium ions served as initiators to cross-link alginates for hydrogels formation [99]. Dissolved oxygen experiments showed that the oxygen-releasing performance of the hydrogel peaked in 3 ​h, returning to normoxic levels in 24 ​h. The addition of catalase to the solution could convert the byproduct H_2_O_2_ to oxygen, extending the oxygen-releasing time to 48 ​h. However, the oxygen release kinetics occurred as a short burst, gradually decreasing, and was not sufficiently stable. Although the hydrogel promoted wound healing, the effect was not significant. The authors attributed these findings to acute explosive oxidative stress, which may play a positive role in tissue regeneration. Prafulla et al. encapsulated SPO and CPO in a polymer matrix film composed of PCL and polyvinyl alcohol (PVA) to prepare a multilayer wound dressing, which allowed *in situ* oxygen generation with gradual tissue fluid penetration [100]. In addition, polyvinylidene chloride was used as the outermost layer of the wound dressing, a material with low gas permeability that allowed oxygen to diffuse uni-directionally to the wound site, further improving the utilization efficacy of the oxygen produced. The oxygen-releasing process of the dressing was retained for up to three days, indicating the rational dressing change time observed in porcine full-thickness skin wounds.

However, chronic wounds fail to heal within 3 days, necessitating more frequent dressing changes to solve the issue of insufficient oxygen release time, which could negatively impact wound healing. Developing materials that afford superior on sustained-release may be a feasible strategy to address this challenge. Zhang et al. conducted a comparative study of PCL/CPO oxygen-releasing microspheres prepared using different methods including homogenization, single-nozzle electrospray and coaxial-nozzle electrospray [101]. The microsphere shell was composed of hydrophobic PCL, isolating the CPO particles from the water and markedly reducing their decomposition rate, which yielded microspheres exhibiting a sustained-release property proportional to the thickness of the PCL layer (Fig. 4G、H). Interestingly, the microspheres prepared by the electrospray method had no obvious pores on their surface, when compared with those prepared using the homogenization method. This finding could be explained by the anhydrous nature of the electrospray method, which hindered CPO from reacting with water during fabrication procedures, resulting in no oxygen leakage for pore formation, and further restricting the contact between CPO and water, improving the sustained-release property. Based on dissolved oxygen experiments, the monolayer PCL/CPO microspheres prepared using the electrospray method could maintain the oxygen concentration of the PBS solution above 10% for at least five days under hypoxic conditions. Suvarnapathaki et al. prepared PCL/CPO microparticles by emulsification process and encapsulated the prepared particles in a methacrylate gelatin (GelMA) hydrogel [102]. The experimental group with the highest CPO content maintained the oxygen concentration of the catalase-supplemented solution supplemented above 10% for up to 35 days, exhibiting a superior sustained-release performance. Theoretically, the CPO-mediated water-splitting could generate calcium hydroxide, which would increase the pH and Ca^2+^ content of the solution, inducing possible cytotoxicity. However, the authors detected no significant change in pH or decrease in cell activity during an incubation time of up to 35 days. Possible explanations include the sequestration effect of the PCL layer and the catalase-mediated detoxification effect. It is worth mentioning that the incorporation of solid peroxides impacts on the physicochemical properties of the prepared biomaterials. Akhavan-Kharazian et al. improved the mechanical properties of wound dressings by mixing CPO particles with polymer films based on chitosan and gelatin [103]. This preparation method also improved the dressing performance in terms of wound healing by reducing the excessive swelling rate, as well as and water vapor transmission rate of the wound dressing. Meanwhile, owing to the presence of CPO, the dressing could release oxygen slowly for at least 10 days, maintaining the pO_2_ of the hypoxic solution at approximately 20 ​mmHg, which enhanced the antibacterial effect and accelerated the growth of cultured cells.

### 3.4 Oxygen-generating microorganisms

**Fig. 5:**
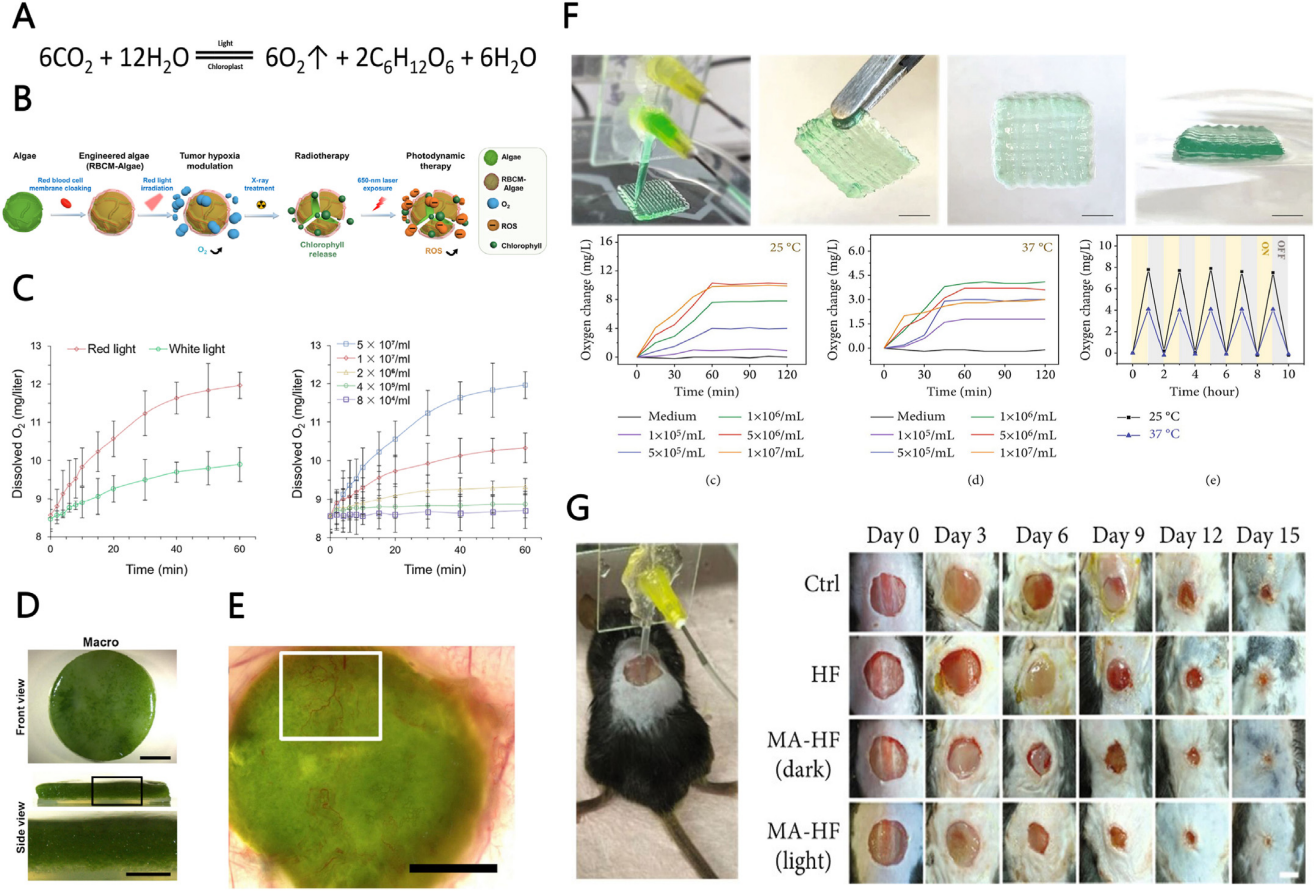
**Microalgae ORBMs. (A)** Photosynthesis. **(B)** Illustrative description of engineered processes and treatments. **(C)** Oxygen-releasing property of the RBCM-Algae under different conditions. **(D)** Fibrin-algae hydrogel scaffolds. **(E)** A detailed analysis of the implanted biomaterial showing high vascularization levels. **(F)** Upper: Photograph of *in situ* 3D-bioprinting photosynthetic Scaffolds. Lower: Photosynthetic oxygen-generating capability of scaffolds under different conditions. **(G)***In situ* bioprinting scaffolds for healing chronic wounds. ORBMs, oxygen-releasing biomaterials; RBCM, red blood cell membrane; SEM, scanning electron microscopy. Reproduced with permission.^[^ [108]^],^ [110,111] Copyright 2020, American Association for the Advancement of Science. Copyright 2015, 2016, Elsevier. Reproduced under terms of the *C*C-BY license [115]. Copyright 2022, Wang et al. published by American Association for the Advancement of Science. (For interpretation of the references to color in this figure legend, the reader is referred to the Web version of this article.)

It is well-established that certain microorganisms can generate oxygen through photosynthesis, including microalgae, the most primitive and largest autotrophic oxygen-generating organism, with 40% of the oxygen on Earth attributed to their photosynthetic activity (Fig. 5A). Numerous studies have reported medical applications of microalgae. Many secondary metabolites of microalgae and their extracts, such as pigments, unsaturated fatty acids, and polysaccharides, exert considerable antioxidant, anti-inflammatory, antimicrobial, and other potential positive effects for wound healing and skin regeneration [104,105]. Many pre-clinical and clinical studies focusing on these active substances as ingredients for wound healing have achieved satisfactory results. A previous study used chlorophyll in microalgae as a photosensitizer, which could heat up under external light, generating ROS, killing bacteria and promoting the healing of infected wounds [106]. As living organisms, microalgae afford good cytocompatibility and no genotoxicity. Based on the active metabolites and autogenous oxygen generation properties, microalgae haves been widely used to study of some oxygen deficiency aggravated diseases, such as tumors, ischemic heart disease, and chronic wounds [107].

Combining microalgae with biomaterials could be a suitable strategy to prepare hydrogels, scaffolds, and dressings for *in situ* autogenous oxygen generation to correct the hypoxic state in chronic wounds. Qiao et al. encapsulated *Chlorella vulgaris* (a type of microalgae) with a red blood cell membrane (RBCM) to improve cytocompatibility (Fig. 5B). Further experiments confirmed that RBCM-Algae did not affect the survival of co-cultured cells even at doses as high as 5 ​× ​10^7^ ​cells/mL [108]. Considering oxygen generation, RBCM-Algae could stably release oxygen under 660 ​nm red light irradiation, with the dissolved oxygen content increasing to 11 ​mg/L in less than 30min (Fig. 5C). Schenck et al. developed a photosynthetic oxygen-generating biomaterial by planting *Chlamydomeae reinhardtii* (a kind of microalgae) in a fibrin hydrogel [109,110]. *C. reinhardtii* proliferated spontaneously in the hydrogel and saturated the oxygen sensor (＞50%) within several days. After grafting the hydrogel into the wound area for five days, the authors detected notable vascular network formation, indicating a positive symbiotic relationship between in the chimeric tissue formed by *C. reinhardtii* and histiocytes (Fig. 5D). After the wound area showed sufficient improvement with hydrogel application, achieving substantial vascularization, unwanted *C. reinhardtii* could be removed using methods such as light deprivation and nontoxic herbicides. Notably, both wound healing and zebrafish injection experiments revealed that the composite hydrogel exhibited low immunogenicity without triggering any severe inflammatory responses in the host, even mice with full immune function. These findings may be partly due to the encapsulation of the fibrin hydrogel. In subsequent experiments, *C. reinhardtii* was transfected with the VEGF gene through genetic engineering to secrete VEGF at the wound site to further promote angiogenesis and wound healing (Fig. 5E) [111]. However, despite increasing the VEGF content, angiogenesis afforded no significant improvement when compared with that of the wild *C. reinhardtii* group. According to the authors, this could be attributed to the multilevel regulation of angiogenesis. Nevertheless, this strategy provided a novel concept for the subsequent research, proposing an “oxygen-releasing bioreactor” based on photosynthetic microalgae. Interestingly, the authors also planted *C. reinhardtii* on commercial surgical sutures, which released recombinant growth factors and oxygen for at least 14 days *in vitro* [112].

Nevertheless, there are still several issues worth exploring regarding this strategy, including the fine regulation of oxygen release kinetics. Furthermore, encapsulation should be considered, in addition to traditional feasible methods such as altering the content of microalgae inoculated and controlling the lighting conditions. Zhong et al. deposited calcium phosphate on the surface of microalgal cells to form a protective mineralization layer [113]. This encapsulation strategy improved microalgae biocompatibility and smoothed oxygen-releasing properties. In contrast, the co-encapsulated polymers could affect the oxygen release behavior of microalgae, which may be attributed to different substrates exerting distinct effects on microalgae proliferation, such as light transmittance and biological adhesion. For example, silk fibroin hydrogels grown with microalgae prolonged the oxygen release time when compared with that of sodium alginate hydrogels [114]. As another major drawback, of the microalgae-based oxygen generation system cannot yield a 24-h oxygen supply without interruption. In the absence of effective light, microalgae respiration competes with tissue cells in the wound area for oxygen, repeatedly disrupting oxygen homeostasis and exacerbating hypoxia. One feasible solution involves the introduction of PFC as a storage container for oxygen, acting as a buffer against changes in oxygen concentration. In addition, the implantation of living organisms into biomaterials presents additional challenges for sterilization and storage [107].

Animal experiments using a microalgae-based oxygen generation system to treat chronic wounds have reported encouraging results. Wang et al. used a coaxial microfluidic-assisted bioprinting device to prepare chimeric microfiber scaffolds with *Chlorella* cells [115]. The outer layer of the fiber was composed of sodium alginate and GelMA copolymer, whereas the inner layer was a solution containing *Chlorella* cells. The scaffolds increased the local oxygen concentration to 3 ​mg/L, maintaining levels for at least 60 ​min at 37 ​°C under external light (Fig. 5F). Furthermore, the oxygen-releasing capacity of prepared scaffolds could be tuned by adjusting the amount of *Chlorella* deposited in fibers and the external lighting conditions. This microfiber scaffold affords a unique advantage, as it could be directly printed and deposited *in situ* at the wound site to accommodate different wound shapes and depths. On application to wounds of diabetic mice, the 15-day wound area of the “scaffold ​+ ​light” group was only 11 ​± ​0.3%, which was significantly narrower than that of the blank control group (Fig. 5G). However, although light treatment could promote collagen deposition and angiogenesis in wounds, the authors failed to note any significant differences in wound closure. Accordingly, *Chlorella* itself may produce bioactive substances equivalent to oxygen to promote wound healing. Chen et al. developed a multi-layer wound patch containing active *Synechococcus* (a type of microalgae) with a hydrophilic inner layer, allowing the patch to improve dissolved oxygen delivery to the wound site [116]. The nutrient carbon source required for microalgae survival could impact oxygen-generating capacity by regulating the metabolic activity of microalgae. Notably, the patch could deliver dissolved oxygen to the skin 100 times more efficiently than topical gaseous oxygen therapy. When applied to diabetic wounds, the patch could convert chronic wounds into acute wounds and shorten the wound healing time to that of normal wounds (45% in 6 days and complete healing in 12 days). In addition, the authors confirmed that the patch could significantly promote the survival of the grafted skin flap, with a mean necrosis rate of only 18.0%, compared with respectively 51.4, 58.0, and 97.5 in the non-diabetic control, topical gaseous oxygen, and diabetic control groups, respectively. Given that topical gaseous oxygen therapy has been applied in the clinical treatment of chronic wounds and flap transplantation, these experimental results might be valuable reference points.

### 3.5 Other oxygen carriers

**Microbubbles (MBs)** are microparticles formed by encapsulating gas in a film shell using lipids, proteins, surfactants, or high molecular weight polymers. In clinical settings, MBs have been used for decades as ultrasound contrast agents and drug delivery systems [117,118]. Ultrasound is closely associated with MBs, not only serving as a synthesis method for MBs but also enabling the achievement of controlled-release properties by destroying bubble stability via the cavitation effect. Conversely, MBs containing oxygen can be a viable option for treating hypoxic diseases such as tumors by combining technologies such as targeted drug delivery [119,120]. Gaseous oxygen in the MBs traverses the physical barrier of the outer shell and diffuses into the surroundings, predominantly driven by the concentration gradient. The stability and oxygen-releasing properties of MBs are largely dependent on their shell materials and oxygen permeability. The reported oxygen release time can vary from minutes to weeks, depending on the material used and preparation method [118]. Khan et al. utilized composite lipids, primarily 1,2-distearoyl-*sn*-glycero-3-phosphocholine (DSPC), to synthesize nanoscale MBs, which could retain more than half of the oxygen load within 3 weeks [121]. The prepared MBs could effectively reverse the hypoxic conditions of *in vitro* cell culture. Conversely, Swanson et al. prepared oxygen-releasing MBs using bovine serum albumin, which retained more than 50% oxygen load for 12 days *in vitro.* [122] Farris et al. prepared a 3D-printed ORBM by embedding PVA and PLGA biodegradable microtanks into PCL scaffolds [123]. After pure oxygen loading at 300–500 psi for one week, the microtanks could release oxygen for 8 ​h when placed outside. In addition, short-term oxygen delivery by microtanks enhanced human adipose-derived stem cell-mediated bone regeneration.

**Cyclodextrins (CDs)** are stereoscopic annular conical oligosaccharides with amphiphilic properties, which are derived from starch hydrolysis. Major natural CDs are named α, β, and γ, comprising six, seven and eight glucopyranose units, respectively. CDs can be used to encapsulate guest molecules of the corresponding size owing to their typical hollow cylindrical structure [124]. This embedding effect renders CDs suitable for application as drug delivery systems, increasing the solubility, stability, and release characteristics of drugs. CDs are widely used in the pharmaceutical field [125]. CDs can be become effective oxygen carriers by embedding oxygen as a guest molecule. Cavalli et al. developed a CD-based nanosponge (a nanostructure prepared by cross-linking CDs, that could stably maintain an oxygen concentration above 5 ​mg/L for 48 ​h under anoxic conditions [126]. Meanwhile, given that CDs are oligomeric saccharides, the inherent superior biocompatibility of the nanosponge was confirmed using *in vitro* cell cultures. Moreover, CD-mediated oxygen delivery could also be used to treat myocardial infarction by rectifying myocardial damage induced by ischemia-reperfusion injury [127]. Overall, these findings indicate that CDs are promising oxygen carriers.

**Endoperoxides** are compounds produced by some cyclic organic molecules, such as pyridine, by forming a peroxy bond within the molecule and undergoing decomposition spontaneously or under heating and other conditions to generate singlet oxygen (^1^O_2_), thus serving as a special oxygen source [128]. Benz et al. developed diverse pyridone compounds with varying degrees of methyl substitution, thereby impacting the *in vitro* half-life and oxygen release properties [129]. Pyridone with three substituted methyl groups has a half-life of 15 ​h for optimal stability and oxygen release when compared with 0.5 ​h for those without methyl groups. However, it should be noted that endoperoxides release oxygen molecules as singlet oxygen (a type of ROS), which is more oxidative than ordinary oxygen molecules. In the presence of vitamin C as a singlet oxygen quencher to remove oxidative toxicity, pyridone endoperoxide could promote cell survival and proliferation under strict hypoxic conditions.

### 3.6 Brief summary

The preceding section of the text describes and discusses the ORBMs across different categories. Table 2 summarizes and compares ORBM characteristics. Furthermore, considering the treatment of diabetic wounds as an example, some experimental methods and specific data of various types of ORBMs are summarized in Table 3 to afford a more intuitive comparison.

**Table 2:** Comparison of various oxygen-releasing biomaterials.

	Hb	PFC	H_2_O_2_	Solid peroxides	Microalgae
Physical and chemical properties	Biomacromolecule; variable oxygen affinities mediated by synergistic and allosteric effects	Hydrophobicity and lipophilicity liquid; high oxygen solubility	Hydrophilic liquid; strong oxidizing property; spontaneous or catalytic decomposition (catalase or metal ions like Mn2+)	Solid; decomposition in contact with water	Autotrophic microorganisms
Mechanisms	Coordinate bond with ferrous ions (Fe^2+^)	Intermolecular forces (van der Waals forces)	Disproportionate decomposition	Hydrolysis	Photosynthesis
Preparation methods	Molecule modification, grafting, hydrogel encapsulation, microsphere encapsulation, electrostatic spraying, needs to be saturated with pure oxygen	Emulsification, grafting, hydrogel encapsulation, microsphere encapsulation, needs to be saturated with pure oxygen	Hydrogel encapsulation, microsphere encapsulation, complexation, *in situ* generation	Hydrogel encapsulation, microsphere encapsulation, electrospray, emulsification	Hydrogel encapsulation, microsphere encapsulation, planted in sutures，three-dimensional printing scaffold
Oxygen payload	Lowest	Low	High	High	Highest
Features of oxygen release kinetics: speed and time	Physical diffusion, first fast then slow, short release time (usually within 24 ​h); responsive controlled-release (temperature, pO_2_); recyclable release	Physical diffusion, first fast, then slow; short release time (usually within 48 ​h), responsive controlled-release (temperature, ultrasound); recyclable release	Chemical reaction; long release time (within weeks); burst release without encapsulation; stable and sustained-release	Chemical reaction; long release time (within weeks); burst release without encapsulation; stable and sustained-release	Biochemical reaction, long release time (infinite as long as microalgae lives); responsive controlled-release (light) and stable sustained-release, but not ceaseless
Advantages	Biological origin (natural human protein), easy availability; can be used as enzymes	Good biocompatibility and chemical inertness; can be used as ultrasound agents	Easy availability; may have some positive impacts at low dosage	Easy availability, relatively high oxygen payload	Generates other bioactive ingredients; highly controllable oxygen-release; can be combined with genetic engineering
Disadvantages	Toxic after decomposition (causing vasoconstriction and oxidative damage by generating ROS)	May cause some adverse effects like flu-like symptoms	Can cause oxidative damage and induce cell apoptosis at a dosage of > 0.04 ​mM	Generates toxic by-products (metal ions and increased pH) and ROS	Cannot generate oxygen continuously for over 12 ​h, additional difficulties regarding storage and sterilization
Ref.	[[51], [52], [53], [54], [55], [56], [57], [58], [59], [60], [61], [62], [63], [64], [65], [66], [67], [68]]	[[69], [70], [71], [72], [73], [74], [75], [76], [77], [78], [79], [80], [81]]	[[82], [83], [84], [85], [86], [87], [88], [89]]	[[90], [91], [92], [93], [94], [95], [96], [97]]	[[98], [99], [100], [101], [102], [103], [104], [105], [106], [107], [108], [109], [110]]

Hb, hemoglobin; PFC, perfluorocarbons; ROS, reactive oxygen species.

**Table 3:** Oxygen-releasing biomaterials for chronic wounds.

ORBMs		Experimental subjects	Estimated average O_2_ concentration	Applying methods	Positive results	Healing ratio （Day: %, vs control）	Ref.
Oxygen carriers	Other constituents/functions						
Hb Hydrogel	HA-DA: Hydrogel matrix, anti-inflammatory PDA: Antioxidant MXene: Photothermal conversion, antimicrobial	Diabetic rats (induced by STZ, same as below) Full-thickness infected wounds Diameter: 6 ​mm	22.7 ​mg/L in 10min with NIR	Hydrogel ​+ ​808 ​nm NIR	Hemostasis， cell migration，collagen deposition, anti-inflammatory (TNF-α↓, IL-10↓, M1/M2↓), angiogenesis (EGF↑, VEGF↑, CD31↑), antimicrobial, antioxidation	6th: 86% vs 31% 9th: 99% vs58%	[67]
PFC Emulsion	HAS: Surfactant	Diabetic rats Forepaw wounds Diameter: 5 ​mm	7 ​mg/L in 40min with rESW	Intravenous injection of PFC emulsion ​+ ​pure oxygen breath for 20 ​min ​+ ​rESW for 20 ​min	Increase blood microcirculation, reduce oxidative stress	4th: 43% vs 19% 8th: 57% vs 36%	[79]
PFC Hydrogel dressing	Chitosan: Hydrogel matrix PHMB: Antimicrobial EGF: Cell proliferation	Diabetic rats Full-thickness wounds Diameter: 8 ​mm	Able to deliver O_2_	Hydrogel dressing; changed every 72 ​h	Anti-inflammatory (IL-8↓), antimicrobial, collagen deposition and maturity, re-epithelialization	9th: 74% vs 65% 15th: 95% vs 81%	[81]
H_2_O_2_ Hydrogel	PDA/AM: Hydrogel matrix PDA NPs: Photothermal conversion, antimicrobial GOx & CAT: Break down glucose and generate O_2_	Diabetic mice	18.6 ​mg/L	Hydrogel ​+ ​808 ​nm NIR for 10 ​min	Hemostasis, antimicrobial, lower blood sugar, angiogenesis, anti-inflammatory, collagen-deposition	7th: 93% vs 83% 14th: 100% vs 99%	[89]
CaO_2_ Hydrogel	Alginate: Hydrogel matrix	Normal mice Full-thickness wounds Diameter: 8 ​mm	35% in 24 ​h	Hydrogel	Tissue growth and remodeling mediated by acute hyperoxic stress	7th: 83% vs 67% 14th: 100% vs 99%	[93]
Microalgae Hydrogel scaffold	GelMA and Alginate: Hydrogel matrix	Diabetic mice Full-thickness wounds Diameter: 10 ​mm	4.1 ​mg/L in 2 ​h with light	*In situ* bioprinting of hydrogel ​+ ​UV (for gelation), exposed to the LED light for 2 ​h every three days	Cell migration, collagen deposition, angiogenesis (CD31↑), tissue remodeling, re-epithelialization	9th: 75% vs 48% 15th: 99% vs 87%	[109]
Microalgae Hydrogel patch	Airtight outer layer Alginate: Hydrogel matrix Hydrophilic inner layer	Diabetic mice Full-thickness wounds Diameter: 10 ​mm	19.2 ​mg/L (600 ​μM) in 1 ​h with light	Hydrogel patch, exposed to NIR LED for 60 ​min, changed every day	Cell migration, anti-inflammatory (NF-κB↓, iNOS↓), angiogenesis (VEFGR1↑), collagen deposition, re-epithelialization	6th: 45% vs 20% 12th: 100% vs 70%	[110]

CAT, catalase; EGF, epidermal growth factor; GelMA, gelatin methacrylate; GOx, glucose oxidase; Hb, hemoglobin; IL-10, interleukin-10; iNOS, inducible nitric oxide synthase; NF-κβ, nuclear factor-kappa beta; NIR, near-infrared; NPs, nanoparticles; PDA, polydopamine; PDA/AM, polydopamine/acrylamide; PFC, perfluorocarbon; rESW, radial extracorporeal shock wave STZ, streptozocin; TNF-α, tumor necrosis factor-α; VEGF, vascular endothelial growth factor; VEGFR1, vascular endothelial growth factor receptor 1.

It is worth mentioning that O2 concentration and exposure time are the two most critical indicators in reoxygenation therapy. However, although many studies concerning ORBMs have achieved satisfactory positive results, there is a lack of detailed quantification of these two therapeutic indicators in a unified framework, and they vary considerably from study to study, such as those data presented in the " estimation of average O2 concentration " and " applying methods " columns of Table 3. This may be due to the insufficient corresponding basic research on the deep mechanism between O2 metabolism and wound healing, thus being unable to provide relatively consistent theoretical guidance. Yet it is clear that ORBM-based reoxygenation therapy is certainly beneficial to wound healing, and the specific mechanism remains one of the future trends regarding ORBMs.

The hypoxic microenvironment in chronic wounds remains a challenge that hinders wound healing; meanwhile, the curative effects of supplementing exogenous oxygen have been positively affirmed. To treat chronic wounds with ORBMs, oxygen should be employed as the main therapeutic drug, while various biomaterials can be employed as carriers to control the speed, dose, and time of oxygen release, as well as to provide additional functions. For fabricating ORBMs, a major consideration involves clarifying the specific requirements of potential application scenarios, which would markedly guide and provide reference values for material preparation and optimization, facilitating the therapeutic relevance for chronic wounds and yielding more positive outcomes.

## 4 Outlook

In the next ten years, it is foreseeable the treatment of chronic wounds will remain an intractable issue plaguing medical and healthcare systems. Various preclinical, clinical, and commercial studies have achieved satisfying results regarding ORBMs for chronic wounds, affording a feasible strategy with considerable potential for future applications. In the field of wound therapy, one goal about is to standardize therapy from the perspective of clinical practice, establishing a standard efficacy evaluation system mainly based on treatment time and dose, positive outcome indicators and possible toxic and side effects. Three main directions should be considered for future research: ⅰ) Materials. Most of the current oxygen-releasing patterns of ORBMs are single-switch and relatively passive, which makes it difficult to dynamically adjust and maintain the local O2 concentration in the wound area. Therefore, one of the future trends is to enhance the material performance for the precise control of oxygen-releasing time and dose throughout the optimization of design and preparation, in order to better meet the needs of application scenarios; ⅱ) Mechanisms. As mentioned earlier, although O2 plays an important role in wound healing, like the two sides of the coin, the biological effect of O2 metabolism on tissues and cells is twofold. A measure of hypoxia level can promote tissue regeneration yet severe hyperoxia hinder the cell survival and the proliferative phase switch. Therefore, basic studies are needed to further elucidate the deep molecular regulation and complete gene expression mechanisms of O2 metabolism to determine the optimal O2 concentration in the whole timeline of wound healing. The basic progress will provide a solid theoretical footstone for subsequent large-scale clinical application of ORBMs; ⅲ) Clinic. The ultimate goal of ORBMs always heads to the clinical applications. Although ORBMs are currently achieving satisfactory outcomes in either laboratory and animal models, their clinical applications are still very limited and demand further validation in human. In addition, further clarification is needed to illustrate the specific best way to apply ORBMs in the clinical practice, such as hydrogels, microspheres, liposomes, etc. We should strengthen the clinical translation of relevant research results and optimize or even alter existing clinical reoxygenation therapies, potentially generating outstanding economic and health benefits.

## Declaration of competing interest

The authors declare that they have no known competing financial interests or personal relationships that could have appeared to influence the work reported in this paper.

## Data Availability

Data will be made available on request.

## References

[1] Graves N., Phillips C.J., Harding K. (2022). A narrative review of the epidemiology and economics of chronic wounds. Br. J. Dermatol..

[2] Olsson M., Jarbrink K., Divakar U., Bajpai R., Upton Z., Schmidtchen A., Car J. (2019). The humanistic and economic burden of chronic wounds: a systematic review. Wound Repair Regen..

[3] Kolimi P., Narala S., Nyavanandi D., Youssef A.A.A., Dudhipala N. (2022). Innovative treatment strategies to accelerate wound healing: trajectory and recent advancements. Cells.

[4] Eisenbud D.E. (2012). Oxygen in wound healing: nutrient, antibiotic, signaling molecule, and therapeutic agent. Clin. Plast. Surg..

[5] Carter M.J., Frykberg R.G., Oropallo A., Sen C.K., Armstrong D.G., Nair H.K.R., Serena T.E. (2023). Efficacy of topical wound oxygen therapy in healing chronic diabetic foot ulcers: systematic review and meta-analysis. Adv. Wound Care..

[6] Hajhosseini B., Kuehlmann B.A., Bonham C.A., Kamperman K.J., Gurtner G.C. (2020). Hyperbaric oxygen therapy: descriptive review of the technology and current application in chronic wounds. Plast Reconstr Surg Glob Open.

[7] Frykberg R.G. (2021). Topical wound oxygen therapy in the treatment of chronic diabetic foot ulcers. Medicina.

[8] Tejada S., Batle J.M., Ferrer M.D., Busquets-Cortes C., Monserrat-Mesquida M., Nabavi S.M., Del Mar Bibiloni M., Pons A., Sureda A. (2019). Therapeutic effects of hyperbaric oxygen in the process of wound healing. Curr. Pharmaceut. Des..

[9] Ortega M.A., Fraile-Martinez O., Garcia-Montero C., Callejon-Pelaez E., Saez M.A., Alvarez-Mon M.A., Garcia-Honduvilla N., Monserrat J., Alvarez-Mon M., Bujan J., Canals M.L. (2021). A general overview on the hyperbaric oxygen therapy: applications, mechanisms and translational opportunities. Medicina.

[10] Erdem A., Haghniaz R., Ertas Y.N., Sangabathuni S.K., Nasr A.S., Swieszkowski W., Ashammakhi N. (2022). Methods for fabricating oxygen releasing biomaterials. J. Drug Target..

[11] Haalboom M. (2018). Chronic wounds: innovations in diagnostics and therapeutics. Curr. Med. Chem..

[12] Cheng B., Fu X. (2018). The focus and target: angiogenesis in refractory wound healing. Int. J. Low. Extrem. Wounds.

[13] Wallace L.A., Gwynne L., Jenkins T. (2019). Challenges and opportunities of pH in chronic wounds. Ther. Deliv..

[14] Gordillo G.M., Sen C.K. (2003). Revisiting the essential role of oxygen in wound healing. Am. J. Surg..

[15] Gottrup F., Firmin R., Rabkin J., Halliday B.J., Hunt T.K. (1987). Directly measured tissue oxygen tension and arterial oxygen tension assess tissue perfusion. Crit. Care Med..

[16] Tandara A.A., Mustoe T.A. (2004). Oxygen in wound healing--more than a nutrient. World J. Surg..

[17] Remensnyder J.P., Majno G. (1968). Oxygen gradients in healing wounds. Am. J. Pathol..

[18] Chen Z., Liu M., Li L., Chen L. (2018). Involvement of the Warburg effect in non-tumor diseases processes. J. Cell. Physiol..

[19] Vaupel P., Multhoff G. (2021). Revisiting the Warburg effect: historical dogma versus current understanding. J. Physiol..

[20] Guo Y., Tan J., Miao Y., Sun Z., Zhang Q. (2019). Effects of microvesicles on cell apoptosis under hypoxia. Oxid. Med. Cell. Longev..

[21] Ravanan P., Srikumar I.F., Talwar P. (2017). Autophagy: the spotlight for cellular stress responses. Life Sci..

[22] Buch P.J., Chai Y., Goluch E.D. (2021). Bacterial chatter in chronic wound infections. Wound Repair Regen..

[23] Gompelman M., van Asten S.A.V., Peters E.J.G. (2016). Update on the role of infection and biofilms in wound healing: pathophysiology and treatment. Plast. Reconstr. Surg..

[24] Thomas D.C. (2017). The phagocyte respiratory burst: historical perspectives and recent advances. Immunol. Lett..

[25] Dunnill C., Patton T., Brennan J., Barrett J., Dryden M., Cooke J., Leaper D., Georgopoulos N.T. (2017). Reactive oxygen species (ROS) and wound healing: the functional role of ROS and emerging ROS-modulating technologies for augmentation of the healing process. Int. Wound J..

[26] Allen D.B., Maguire J.J., Mahdavian M., Wicke C., Marcocci L., Scheuenstuhl H., Chang M., Le A.X., Hopf H.W., Hunt T.K. (1997). Wound hypoxia and acidosis limit neutrophil bacterial killing mechanisms. Arch. Surg..

[27] Schreml S., Szeimies R.M., Prantl L., Karrer S., Landthaler M., Babilas P. (2010). Oxygen in acute and chronic wound healing. Br. J. Dermatol..

[28] Siddiqui A., Galiano R.D., Connors D., Gruskin E., Wu L., Mustoe T.A. (1996). Differential effects of oxygen on human dermal fibroblasts: acute versus chronic hypoxia. Wound Repair Regen..

[29] Tuderman L., Myllyla R., Kivirikko K.I. (1977). Mechanism of the prolyl hydroxylase reaction. 1. Role of co-substrates. Eur. J. Biochem..

[30] Myllyla R., Tuderman L., Kivirikko K.I. (1977). Mechanism of the prolyl hydroxylase reaction. 2. Kinetic analysis of the reaction sequence. Eur. J. Biochem..

[31] Prockop D.J., Kivirikko K.I., Tuderman L., Guzman N.A. (1979). The biosynthesis of collagen and its disorders (first of two parts). N. Engl. J. Med..

[32] Tirpe A.A., Gulei D., Ciortea S.M., Crivii C., Berindan-Neagoe I. (2019). Hypoxia: overview on hypoxia-mediated mechanisms with a focus on the role of HIF genes. Int. J. Mol. Sci..

[33] Manalo D.J., Rowan A., Lavoie T., Natarajan L., Kelly B.D., Ye S.Q., Garcia J.G., Semenza G.L. (2005). Transcriptional regulation of vascular endothelial cell responses to hypoxia by HIF-1. Blood.

[34] Sunkari V.G., Lind F., Botusan I.R., Kashif A., Liu Z.J., Yla-Herttuala S., Brismar K., Velazquez O., Catrina S.B. (2015). Hyperbaric oxygen therapy activates hypoxia-inducible factor 1 (HIF-1), which contributes to improved wound healing in diabetic mice. Wound Repair Regen..

[35] Isaacs J.S., Jung Y.J., Mimnaugh E.G., Martinez A., Cuttitta F., Neckers L.M. (2002). Hsp90 regulates a von Hippel Lindau-independent hypoxia-inducible factor-1 alpha-degradative pathway. J. Biol. Chem..

[36] Semadi N.I. (2019). The role of VEGF and TNF-alpha on epithelialization of diabetic foot ulcers after hyperbaric oxygen therapy. Open Access Maced J Med Sci.

[37] Sen C.K. (2009). Wound healing essentials: let there be oxygen. Wound Repair Regen..

[38] Nit K., Tyszka-Czochara M., Bobis-Wozowicz S. (2021). Oxygen as a master regulator of human pluripotent stem cell function and metabolism. J. Personalized Med..

[39] Nishizaka T., Nomura T., Higuchi K., Takemura A., Ishihara A. (2018). Mild hyperbaric oxygen activates the proliferation of epidermal basal cells in aged mice. J. Dermatol..

[40] Huang X., Liang P., Jiang B., Zhang P., Yu W., Duan M., Guo L., Cui X., Huang M., Huang X. (2020). Hyperbaric oxygen potentiates diabetic wound healing by promoting fibroblast cell proliferation and endothelial cell angiogenesis. Life Sci..

[41] Roy S., Khanna S., Bickerstaff A.A., Subramanian S.V., Atalay M., Bierl M., Pendyala S., Levy D., Sharma N., Venojarvi M., Strauch A., Orosz C.G., Sen C.K. (2003). Oxygen sensing by primary cardiac fibroblasts: a key role of p21(Waf1/Cip1/Sdi1). Circ. Res..

[42] Sander A.L., Henrich D., Muth C.M., Marzi I., Barker J.H., Frank J.M. (2009). In vivo effect of hyperbaric oxygen on wound angiogenesis and epithelialization. Wound Repair Regen..

[43] Ortiz-Prado E., Dunn J.F., Vasconez J., Castillo D., Viscor G. (2019). Partial pressure of oxygen in the human body: a general review. Am J Blood Res.

[44] Singer M., Young P.J., Laffey J.G., Asfar P., Taccone F.S., Skrifvars M.B., Meyhoff C.S., Radermacher P. (2021). Dangers of hyperoxia. Crit. Care.

[45] Thom S.R. (2011). Hyperbaric oxygen: its mechanisms and efficacy. Plast. Reconstr. Surg..

[46] Thackham J.A., McElwain D.L., Long R.J. (2008). The use of hyperbaric oxygen therapy to treat chronic wounds: a review. Wound Repair Regen..

[47] De Wolde S.D., Hulskes R.H., Weenink R.P., Hollmann M.W., Van Hulst R.A. (2021). The effects of hyperbaric oxygenation on oxidative stress, inflammation and angiogenesis. Biomolecules.

[48] Cholewka A., Knefel G., Stanek A., Kawecki M., Nowak M., Aleksander S., Zofia D. (2012). Thermal imaging and TC oximetry measurements of hyperbaric oxygen therapy (HBO) effects on trophic ulceration of the crura. J. Therm. Anal. Calorim..

[49] Ruzicka J., Dejmek J., Bolek L., Benes J., Kuncova J. (2021). Hyperbaric oxygen influences chronic wound healing - a cellular level review. Physiol. Res..

[50] Glik J., Cholewka A., Stanek A., Englisz B., Sieron K., Mikus-Zagorska K., Knefel G., Nowak M., Kawecki M. (2019). Thermal imaging and planimetry evaluation of the results of chronic wounds treatment with hyperbaric oxygen therapy. Adv. Clin. Exp. Med..

[51] Vivcharenko V., Przekora A. (2021). Modifications of wound dressings with bioactive agents to achieve improved pro-healing properties. Applied Sciences-Basel.

[52] James C.V., Park S.Y., Alabi D., Lantis J.C. (2021). Effect of topical oxygen therapy on chronic wounds. Surg. Technol. Int..

[53] Kaufman H., Gurevich M., Tamir E., Keren E., Alexander L., Hayes P. (2018). Topical oxygen therapy stimulates healing in difficult, chronic wounds: a tertiary centre experience. J. Wound Care.

[54] Otaviano M.H., Salles M., Ching T.H., Dettoni J.L., Coulibaly I.G.S., Fukunaga E.T., Gamba M.A., Moraes J.C. (2021). Topical Oxygen Jet Therapy (TOJT) for treating infected chronic surgical wounds. Braz. J. Infect. Dis..

[55] Kasprzyk-Kucewicz T., Cholewka A., Englisz-Jurgielewicz B., Mucha R., Relich M., Kawecki M., Sieron K., Onak P., Stanek A. (2021). Thermal effects of topical hyperbaric oxygen therapy in hard-to-heal wounds-A pilot study. Int. J. Environ. Res. Publ. Health.

[56] Jebril W., Nowak M., Palin L., Nordgren M., Bachar-Wikstrom E., Wikstrom J.D. (2022). Topical oxygen treatment relieves pain from hard-to-heal leg ulcers and improves healing: a case series. J. Wound Care.

[57] Ahmed M.H., Ghatge M.S., Safo M.K. (2020). Hemoglobin: structure, function and allostery. Subcell. Biochem..

[58] Faggiano S., Ronda L., Bruno S., Abbruzzetti S., Viappiani C., Bettati S., Mozzarelli A. (2022). From hemoglobin allostery to hemoglobin-based oxygen carriers. Mol. Aspect. Med..

[59] Bellelli A., Tame J.R.H. (2022). Hemoglobin allostery and pharmacology. Mol. Aspect. Med..

[60] Gell D.A. (2018). Structure and function of haemoglobins. Blood Cells Mol. Dis..

[61] Sheshadri P., Abraham J. (2012). Antimicrobial properties of hemoglobin. Immunopharmacol. Immunotoxicol..

[62] Bulters D., Gaastra B., Zolnourian A., Alexander S., Ren D., Blackburn S.L., Borsody M., Dore S., Galea J., Iihara K., Nyquist P., Galea I. (2018). Haemoglobin scavenging in intracranial bleeding: biology and clinical implications. Nat. Rev. Neurol..

[63] di Masi A., De Simone G., Ciaccio C., D'Orso S., Coletta M., Ascenzi P. (2020). Haptoglobin: from hemoglobin scavenging to human health. Mol. Aspect. Med..

[64] Manning J.M., Manning L.R., Dumoulin A., Padovan J.C., Chait B. (2020). Embryonic and fetal human hemoglobins: structures, oxygen binding, and physiological roles. Subcell. Biochem..

[65] Ozcelik H., Batool F., Corre M., Garlaschelli A., Conzatti G., Stutz C., Petit C., Delpy E., Zal F., Leize-Zal E., Huck O. (2021). Characterization of a hyaluronic acid-based hydrogel containing an extracellular oxygen carrier (M101) for periodontitis treatment: an in vitro study. Int. J. Pharm..

[66] Rousselot M., Delpy E., Drieu La Rochelle C., Lagente V., Pirow R., Rees J.F., Hagege A., Le Guen D., Hourdez S., Zal F. (2006). Arenicola marina extracellular hemoglobin: a new promising blood substitute. Biotechnol. J..

[67] Paciello A., Amalfitano G., Garziano A., Urciuolo F., Netti P.A. (2016). Hemoglobin-conjugated gelatin microsphere as a smart oxygen releasing biomaterial. Adv Healthc Mater.

[68] Wang Y., Zhang S., Zhang J., Yu W.L., Gao D.W., Wang Q., You G.X., Hu T., Zhao L., Zhou H. (2017). Structural, functional and physiochemical properties of dextran-bovine hemoglobin conjugate as a hemoglobin-based oxygen carrier. Process Biochem..

[69] Wang Q., Zhang R., Lu M., You G., Wang Y., Chen G., Zhao C., Wang Z., Song X., Wu Y., Zhao L., Zhou H. (2017). Bioinspired polydopamine-coated hemoglobin as potential oxygen carrier with antioxidant properties. Biomacromolecules.

[70] Wang Q., Zhang R., You G., Hu J., Li P., Wang Y., Zhang J., Wu Y., Zhao L., Zhou H. (2018). Influence of polydopamine-mediated surface modification on oxygen-release capacity of haemoglobin-based oxygen carriers. Artif. Cells, Nanomed. Biotechnol..

[71] Centis V., Proulx P., Vermette P. (2011). PEGylated liposomes encapsulating human hemoglobin enhance oxygen transfer and cell proliferation while decreasing cell hypoxia in fibrin. Biochem. Eng. J..

[72] Liu X., Jansman M.M.T., Thulstrup P.W., Mendes A.C., Chronakis I.S., Hosta-Rigau L. (2020). Low-fouling electrosprayed hemoglobin nanoparticles with antioxidant protection as promising oxygen carriers. Macromol. Biosci..

[73] Li Y., Fu R., Duan Z., Zhu C., Fan D. (2022). Artificial nonenzymatic antioxidant MXene nanosheet-anchored injectable hydrogel as a mild photothermal-controlled oxygen release platform for diabetic wound healing. ACS Nano.

[74] Hunt S., Elg F. (2017). The clinical effectiveness of haemoglobin spray as adjunctive therapy in the treatment of chronic wounds. J. Wound Care.

[75] Jagers J., Wrobeln A., Ferenz K.B. (2021). Perfluorocarbon-based oxygen carriers: from physics to physiology. Pflügers Archiv.

[76] Krafft M.P., Riess J.G. (2021). Therapeutic oxygen delivery by perfluorocarbon-based colloids. Adv. Colloid Interface Sci..

[77] Lambert E., Gorantla V.S., Janjic J.M. (2019). Pharmaceutical design and development of perfluorocarbon nanocolloids for oxygen delivery in regenerative medicine. Nanomedicine.

[78] Day R.A., Sletten E.M. (2021). Perfluorocarbon nanomaterials for photodynamic therapy. Curr. Opin. Colloid Interface Sci..

[79] Holman R., Lorton O., Guillemin P.C., Desgranges S., Contino-Pepin C., Salomir R. (2021). Perfluorocarbon emulsion contrast agents: a mini review. Front. Chem..

[80] Fu X.T., Ohta S., Kawakatsu T., Kamihira M., Sakai Y., Ito T. (2022). Bioinspired perfluorocarbon-based oxygen carriers with concave shape and deformable shell. Advanced Materials Technologies.

[81] Niu H., Li C., Guan Y., Dang Y., Li X., Fan Z., Shen J., Ma L., Guan J. (2020). High oxygen preservation hydrogels to augment cell survival under hypoxic condition. Acta Biomater..

[82] Patil P.S., Fountas-Davis N., Huang H., Michelle Evancho-Chapman M., Fulton J.A., Shriver L.P., Leipzig N.D. (2016). Fluorinated methacrylamide chitosan hydrogels enhance collagen synthesis in wound healing through increased oxygen availability. Acta Biomater..

[83] Li S.Y., Chen A.Q., Chen Y.X., Yang Y., Zhang Q.W., Luo S., Ye M.Q., Zhou Y.J., An Y., Huang W., Xuan T.X., Pan Y.X., Xuan X., He H.C., Wu J. (2020). Lotus leaf inspired antiadhesive and antibacterial gauze for enhanced infected dermal wound regeneration. Chem. Eng. J..

[84] Zhang S., Li Z., Wang Q., Liu Q., Yuan W., Feng W., Li F. (2022). An NIR-II photothermally triggered "oxygen Bomb" for hypoxic tumor programmed cascade therapy. Adv. Mater..

[85] Wang S., Yin C., Han X., Guo A., Chen X., Liu S., Liu Y. (2019). Improved healing of diabetic foot ulcer upon oxygenation therapeutics through oxygen-loading nanoperfluorocarbon triggered by radial extracorporeal shock wave. Oxid. Med. Cell. Longev..

[86] Jalani G., Jeyachandran D., Bertram Church R., Cerruti M. (2017). Graphene oxide-stabilized perfluorocarbon emulsions for controlled oxygen delivery. Nanoscale.

[87] Lee Y.H., Lin S.J. (2022). Chitosan/PVA hetero-composite hydrogel containing antimicrobials, perfluorocarbon nanoemulsions, and growth factor-loaded nanoparticles as a multifunctional dressing for diabetic wound healing: synthesis, characterization, and in vitro/in vivo evaluation. Pharmaceutics.

[88] Zhu G., Wang Q., Lu S., Niu Y. (2017). Hydrogen peroxide: a potential wound therapeutic target?. Med. Princ. Pract..

[89] Xiang J., Wan C., Guo R., Guo D. (2016). Is hydrogen peroxide a suitable apoptosis inducer for all cell types?. BioMed Res. Int..

[90] Heo S., Kim S., Kang D. (2020). The role of hydrogen peroxide and peroxiredoxins throughout the cell cycle. Antioxidants.

[91] Abdi S.I., Ng S.M., Lim J.O. (2011). An enzyme-modulated oxygen-producing micro-system for regenerative therapeutics. Int. J. Pharm..

[92] Li Z., Guo X., Guan J. (2012). An oxygen release system to augment cardiac progenitor cell survival and differentiation under hypoxic condition. Biomaterials.

[93] Sarkandi A.F., Montazer M., Rad M.M. (2022). Oxygenated-bacterial-cellulose nanofibers with hydrogel, antimicrobial, and controlled oxygen release properties for rapid wound healing. J. Appl. Polym. Sci..

[94] Huber D., Tegl G., Mensah A., Beer B., Baumann M., Borth N., Sygmund C., Ludwig R., Guebitz G.M. (2017). A dual-enzyme hydrogen peroxide generation machinery in hydrogels supports antimicrobial wound treatment. ACS Appl. Mater. Interfaces.

[95] Wang Q., Qiu W.W., Liu H., Li X.R., Qin X.H., Wang X.L., Yu J.Y., Li B., Li F.X., Huang L.Q., Wu D.Q. (2022). Conductive hydrogel dressings based on cascade reactions with photothermal effect for monitoring and treatment of diabetic wounds. Compos. B Eng..

[96] Rastinfard A., Dalisson B., Barralet J. (2022). Aqueous decomposition behavior of solid peroxides: effect of pH and buffer composition on oxygen and hydrogen peroxide formation. Acta Biomater..

[97] Wang H., Zhao Y., Li T., Chen Z., Wang Y., Qin C. (2016). Properties of calcium peroxide for release of hydrogen peroxide and oxygen: a kinetics study. Chem. Eng. J..

[98] Wang J., Zhu Y., Bawa H.K., Ng G., Wu Y., Libera M., van der Mei H.C., Busscher H.J., Yu X. (2011). Oxygen-generating nanofiber cell scaffolds with antimicrobial properties. ACS Appl. Mater. Interfaces.

[99] Kang J.I., Park K.M., Park K.D. (2019). Oxygen-generating alginate hydrogels as a bioactive acellular matrix for facilitating wound healing. J. Ind. Eng. Chem..

[100] Chandra P.K., Ross C.L., Smith L.C., Jeong S.S., Kim J., Yoo J.J., Harrison B.S. (2015). Peroxide-based oxygen generating topical wound dressing for enhancing healing of dermal wounds. Wound Repair Regen..

[101] Zhang M., Kiratiwongwan T., Shen W. (2020). Oxygen-releasing polycaprolactone/calcium peroxide composite microspheres. J. Biomed. Mater. Res. B Appl. Biomater..

[102] Suvarnapathaki S., Nguyen M.A., Goulopoulos A.A., Lantigua D., Camci-Unal G. (2021). Engineering calcium peroxide based oxygen generating scaffolds for tissue survival. Biomater. Sci..

[103] Akhavan-Kharazian N., Izadi-Vasafi H. (2019). Preparation and characterization of chitosan/gelatin/nanocrystalline cellulose/calcium peroxide films for potential wound dressing applications. Int. J. Biol. Macromol..

[104] de Andrade A.F., Porto A.L.F., Bezerra R.P. (2022). Photosynthetic microorganisms and their bioactive molecules as new product to healing wounds. Appl. Microbiol. Biotechnol..

[105] Miguel S.P., Ribeiro M.P., Otero A., Coutinho P. (2021). Application of microalgae and microalgal bioactive compounds in skin regeneration. Algal Research-Biomass Biofuels and Bioproducts.

[106] Zhao E., Liu H., Jia Y., Xiao T., Li J., Zhou G., Wang J., Zhou X., Liang X.J., Zhang J., Li Z. (2022). Engineering a photosynthetic bacteria-incorporated hydrogel for infected wound healing. Acta Biomater..

[107] Cui H., Su Y., Wei W., Xu F., Gao J., Zhang W. (2022). How microalgae is effective in oxygen deficiency aggravated diseases? A comprehensive review of literature. Int. J. Nanomed..

[108] Qiao Y., Yang F., Xie T., Du Z., Zhong D., Qi Y., Li Y., Li W., Lu Z., Rao J., Sun Y., Zhou M. (2020). Engineered algae: a novel oxygen-generating system for effective treatment of hypoxic cancer. Sci. Adv..

[109] Hopfner U., Schenck T.L., Chavez M.N., Machens H.G., Bohne A.V., Nickelsen J., Giunta R.E., Egana J.T. (2014). Development of photosynthetic biomaterials for in vitro tissue engineering. Acta Biomater..

[110] Schenck T.L., Hopfner U., Chavez M.N., Machens H.G., Somlai-Schweiger I., Giunta R.E., Bohne A.V., Nickelsen J., Allende M.L., Egana J.T. (2015). Photosynthetic biomaterials: a pathway towards autotrophic tissue engineering. Acta Biomater..

[111] Chavez M.N., Schenck T.L., Hopfner U., Centeno-Cerdas C., Somlai-Schweiger I., Schwarz C., Machens H.G., Heikenwalder M., Bono M.R., Allende M.L., Nickelsen J., Egana J.T. (2016). Towards autotrophic tissue engineering: photosynthetic gene therapy for regeneration. Biomaterials.

[112] Centeno-Cerdas C., Jarquin-Cordero M., Chavez M.N., Hopfner U., Holmes C., Schmauss D., Machens H.G., Nickelsen J., Egana J.T. (2018). Development of photosynthetic sutures for the local delivery of oxygen and recombinant growth factors in wounds. Acta Biomater..

[113] Zhong D., Li W., Hua S., Qi Y., Xie T., Qiao Y., Zhou M. (2021). Calcium phosphate engineered photosynthetic microalgae to combat hypoxic-tumor by in-situ modulating hypoxia and cascade radio-phototherapy. Theranostics.

[114] Fu Y., Xie X., Wang Y., Liu J., Zheng Z., Kaplan D.L., Wang X. (2021). Sustained photosynthesis and oxygen generation of microalgae-embedded silk fibroin hydrogels. ACS Biomater. Sci. Eng..

[115] Wang X., Yang C., Yu Y., Zhao Y. (2022). In situ 3D bioprinting living photosynthetic scaffolds for autotrophic wound healing. Research.

[116] Chen H., Cheng Y., Tian J., Yang P., Zhang X., Chen Y., Hu Y., Wu J. (2020). Dissolved oxygen from microalgae-gel patch promotes chronic wound healing in diabetes. Sci. Adv..

[117] Klibanov A.L. (2021). Ultrasound contrast: gas microbubbles in the vasculature. Invest. Radiol..

[118] Rudakovskaya P.G., Barmin R.A., Kuzmin P.S., Fedotkina E.P., Sencha A.N., Gorin D.A. (2022). Microbubbles stabilized by protein shell: from pioneering ultrasound contrast agents to advanced theranostic systems. Pharmaceutics.

[119] Jangjou A., Meisami A.H., Jamali K., Niakan M.H., Abbasi M., Shafiee M., Salehi M., Hosseinzadeh A., Amani A.M., Vaez A. (2021). The promising shadow of microbubble over medical sciences: from fighting wide scope of prevalence disease to cancer eradication. J. Biomed. Sci..

[120] Khan M.S., Hwang J., Lee K., Choi Y., Kim K., Koo H.J., Hong J.W., Choi J. (2018). Oxygen-carrying micro/nanobubbles: composition, synthesis techniques and potential prospects in photo-triggered theranostics. Molecules.

[121] Khan M.S., Hwang J., Seo Y., Shin K., Lee K., Park C., Choi Y., Hong J.W., Choi J. (2018). Engineering oxygen nanobubbles for the effective reversal of hypoxia. Artif. Cells, Nanomed. Biotechnol..

[122] Swanson E.J., Mohan V., Kheir J., Borden M.A. (2010). Phospholipid-stabilized microbubble foam for injectable oxygen delivery. Langmuir.

[123] Farris A.L., Lambrechts D., Zhou Y., Zhang N.Y., Sarkar N., Moorer M.C., Rindone A.N., Nyberg E.L., Perdomo-Pantoja A., Burris S.J., Free K., Witham T.F., Riddle R.C., Grayson W.L. (2022). 3D-printed oxygen-releasing scaffolds improve bone regeneration in mice. Biomaterials.

[124] Varan G., Varan C., Erdogar N., Hincal A.A., Bilensoy E. (2017). Amphiphilic cyclodextrin nanoparticles. Int. J. Pharm..

[125] Lachowicz M., Stanczak A., Kolodziejczyk M. (2020). Characteristic of cyclodextrins: their role and use in the pharmaceutical technology. Curr. Drug Targets.

[126] Cavalli R., Akhter A.K., Bisazza A., Giustetto P., Trotta F., Vavia P. (2010). Nanosponge formulations as oxygen delivery systems. Int. J. Pharm..

[127] Femmino S., Penna C., Caldera N., Dhakar D., Cau P., Pagliaro R., Trotta Cavalli F., F. Bessone, F. (2018). Alpha-Cyclodextrin and alpha-cyclodextrin polymers as oxygen nanocarriers to limit hypoxia/reoxygenation injury: implications from an in vitro model. Polymers.

[128] Changtong C.C.W., Carney D.W., Luo L., Zoto C.A., Lombardi J.L., Connors R.E. (2013). A porphyrin molecule that generates, traps, stores, and releases singlet oxygen. J. Photochem. Photobiol. Chem..

[129] Benz S., Notzli S., Siegel J.S., Eberli D., Jessen H.J. (2013). Controlled oxygen release from pyridone endoperoxides promotes cell survival under anoxic conditions. J. Med. Chem..

